# Bio-Inspired Functional Surface Texturing by Vibration-Assisted Machining: From Tribological Interfaces to Electrochemical Biosensing

**DOI:** 10.3390/biomimetics11100715

**Published:** 2026-10-08

**Authors:** Saood Ali, Sung-Ho Hong, Moran Xu, Khaled Hamdy, Rakan Albarakati, Abdullah Alhjjaji, Hoong-Pin Lee

**Affiliations:** 1 School of Mechanical Engineering, Yeungnam University, 280, Daehak-ro, Gyeongsan-si 38541, Gyeongsangbuk-do, Republic of Korea; 2 Hubei Key Laboratory of Digital Textile Equipment, Wuhan Textile University, Wuhan 430071, China; 3 Production Engineering and Mechanical Design Department, Faculty of Engineering, Minia University, El-Minia 61111, Egypt; 4 Industrial Engineering Department, College of Engineering and Architecture, Umm Al-Qura University, Makkah 24382, Saudi Arabia; rmbarakati@uqu.edu.sa (R.A.);; 5 Faculty of Engineering & Quantity Surveying, INTI International University, Persiaran Perdana BBN Putra Nilai, Nilai 71800, Negeri Sembilan, Malaysia

**Keywords:** vibration-assisted machining (VAM), biomimetics, bio-inspired surface, hierarchical textures, surface tribology, electrochemical biosensors, micro/nanostructures

## Abstract

**Highlights:**

- VAM enables deterministic fabrication of bio-inspired micro- and hierarchical surface textures.
- Fish-scale and hierarchical textures link natural surface architectures with engineered tribological functions.
- Texture geometry controls friction, fluid transport, wettability, biofouling, and electrochemical activity.
- Hierarchical micro–nano surfaces can combine mechanical durability with enhanced biosensing performance.
- Bio-inspired design and AI-assisted optimization may guide future multifunctional sensing interfaces.

**Abstract:**

Biological surfaces have unique characteristics and properties due to the presence of well-organized micro- and nano-architectures. Fish scales, lotus leaves, shark skin, gecko feet, and insect wings are prime examples of how hierarchical and directional features can control the interfacial interactions which regulate the friction, wettability, fluid transport, adhesion, and fouling properties. However, translation of these surface textures onto engineered surfaces requires precision manufacturing methods which are capable of reproducing well-defined textures while also maintaining surface integrity. The present review examines vibration-assisted machining (VAM) as a precision manufacturing approach for producing functional and bio-inspired surface textures and discusses their potential use in tribological and electrochemical biosensing interfaces. VAM can generate deterministic dimples, grooves, crosshatch patterns, fish-scale structures, and hierarchical micro-textures through controlled tool–workpiece kinematics. Particular attention is given to the relationship between texture geometry and function, including lubricant retention, hydrodynamic pressure generation, contact-area reduction, debris entrapment, wettability, and fluid transport. These mechanisms are then coupled to electrochemical sensing where structured interfaces may increase the electrochemically active surface area, facilitate analyte transport and charge transfer, and provide more sites for bioreceptor immobilization. Hierarchical micro–nano-textures are especially attractive as they combine the mechanical functions of micro-scale structures with the interfacial and electrochemical advantages of nano-scale features. The present manuscript further discusses tribochemical stability, biofouling, physiological interfaces, and emerging biosensing applications. Finally, future opportunities are identified in quantitative bio-inspired texture design, AI-assisted inverse design, hierarchical functionalization, scalable manufacturing, and in vivo validation. This perspective positions VAM as a manufacturing bridge between biological surface design principles and multifunctional engineered interfaces for sensing and biomedical applications.

## 1. Introduction

Biological surfaces have evolved highly organized micro- and nano-scale architectures that enable functions that are difficult to reproduce using conventional smooth engineering surfaces [1,2]. The hierarchical roughness of lotus leaves, anisotropic riblet-like features associated with aquatic organisms, overlapping fish-scale geometries, fibrillar gecko foot structures, and multi-scale insect-wing topographies illustrate a common principle that surface characteristics are governed not only by material chemistry but also by geometry, hierarchy, orientation, and spatial organization [3,4,5,6]. These natural structure–function relationships have inspired engineered surfaces for controlling friction, adhesion, wettability, fluid transport, self-cleaning, antifouling, and sensing [1,2,4,5,7].

The increasing demand for rapid healthcare diagnostics, environmental monitoring, and point-of-care (POC) testing has accelerated the development of biosensors that are highly sensitive, selective, compact, and capable of reliable long-term operation [8,9]. Among the available technologies, electrochemical biosensors have attracted considerable attention as they are easy to integrate with electronic devices, relatively inexpensive to fabricate, and well suited for real-time monitoring [10,11]. However, despite these advantages, the overall functional performance of biosensors depends considerably on the characteristics of the working electrode, particularly its surface architecture [12] and topography [13].

This biomimetic perspective is directly relevant to electrochemical biosensors. Biological interfaces regulate fluid transport, molecular interaction, adhesion, and protection through multi-scale surface organization; comparable requirements exist at biosensor electrodes, where analyte transport, biomolecule immobilization, fouling, wettability, and mechanical stability collectively determine sensing performance. Accordingly, the controlled reproduction of selected biological surface principles offers a route from conventional planar electrodes toward multifunctional sensing interfaces in which geometry contributes actively to both interfacial transport and long-term stability.

At the same time, advances in precision manufacturing have led to an increasing attention in vibration-assisted machining (VAM). By superimposing high-frequency vibrations on a conventional milling process, VAM enables the fabrication of well-controlled periodic micro-scale surface textures with high geometric accuracy, while also reducing cutting forces, improving chip evacuation, and minimizing subsurface damage [14,15]. These advantages make VAM a promising alternative for producing textured electrode surface topographies that otherwise rely primarily on more complex and expensive techniques such as lithography and laser surface texturing [16,17].

Surface texturing has been regularly used to improve the tribological performance by modifying interaction between contact surfaces during sliding. Surface textures such as dimples, grooves, and hierarchical patterns can reduce the actual contact area, improve lubricant film formation, trap wear debris, and generate localized hydrodynamic pressure, all of which help in reducing the coefficient of friction and wear [18,19,20]. Furthermore, these surface textures are also beneficial for electrochemical biosensors. By increasing the effective electrode surface area, improving electrolyte retention and analyte transport, and providing more favorable sites for biomolecule immobilization, textured surfaces can significantly enhance biosensor sensitivity and overall sensing performance [21,22].

Although vibration-assisted machining (VAM), surface tribology, biomimetic surface engineering, and electrochemical biosensing have each developed rapidly, the connections among these fields remain insufficiently defined. In particular, limited attention has been given to how biological structure–function principles can be abstracted into deterministic VAM-generated textures and how the resulting surfaces influence mechanical, interfacial, and electrochemical functions. The present review therefore examines VAM from a bio-inspired surface engineering perspective, linking biological design concepts with manufacturable texture geometries, tribological mechanisms, and emerging electrochemical biosensing applications. Particular emphasis is placed on fish-scale, directional, and hierarchical micro–nano-architectures, together with the process–structure–function relationships required for their practical implementation.

## 2. Vibration-Assisted Machining (VAM): Principles and Texture Generation

### 2.1. Fundamentals of Vibration-Assisted Machining

**Figure 1:**
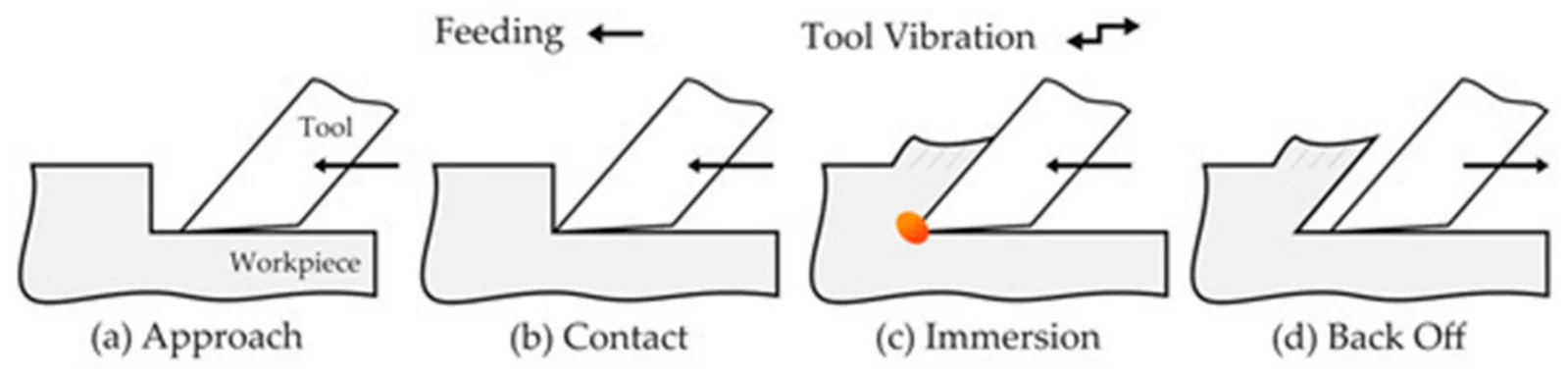
Application of vibration on tool during VAM process (reprinted from Martins et al. [27] with permission from MDPI).

Vibration-assisted machining (VAM) is an advanced machining process in which controlled vibrations are superimposed on the primary cutting motion to improve machining performance [23,24]. During vibration-assisted milling, which is one of the most commonly used methods for surface texturing, the vibrations are superimposed either on the cutting tool (tool-based VAM) or to the workpiece (workpiece-based VAM), and can be applied in one or more directions. As the cutting tool vibrates during cutting motion, contact between the cutting edge and the workpiece is periodically interrupted and this phenomenon is known as tool–workpiece separation (TWS). The intermittent cutting action alters the chip formation, reduces heat generation, and influences the resulting surface topography as compared to conventional milling [25,26]. The basic concept of vibration application during machining is shown in Figure 1.

The vibration frequency used in VAM systems is generally in the ultrasonic frequency range (20–40 kHz) in resonant systems or at lower frequencies (50–2000 Hz) in non-resonant piezo-driven systems [24,28]. Ultrasonic vibration-assisted machining (UVAM) works at a fixed resonant vibration frequency and has high vibration energy, which makes it particularly suitable for machining of hard-to-cut materials such as ceramics, glass, and titanium alloys [29,30]. On the other hand, a non-resonant VAM system provides the option of adjusting the vibration frequency and amplitude independently, which provides greater flexibility in controlling the surface texture geometry. Also, this quality is particularly beneficial for biosensor electrode fabrication, where precise control over surface topography is a very important factor [31,32].

### 2.2. Kinematics of 1D and 2D Vibration-Assisted Machining (VAM)

**Figure 2:**
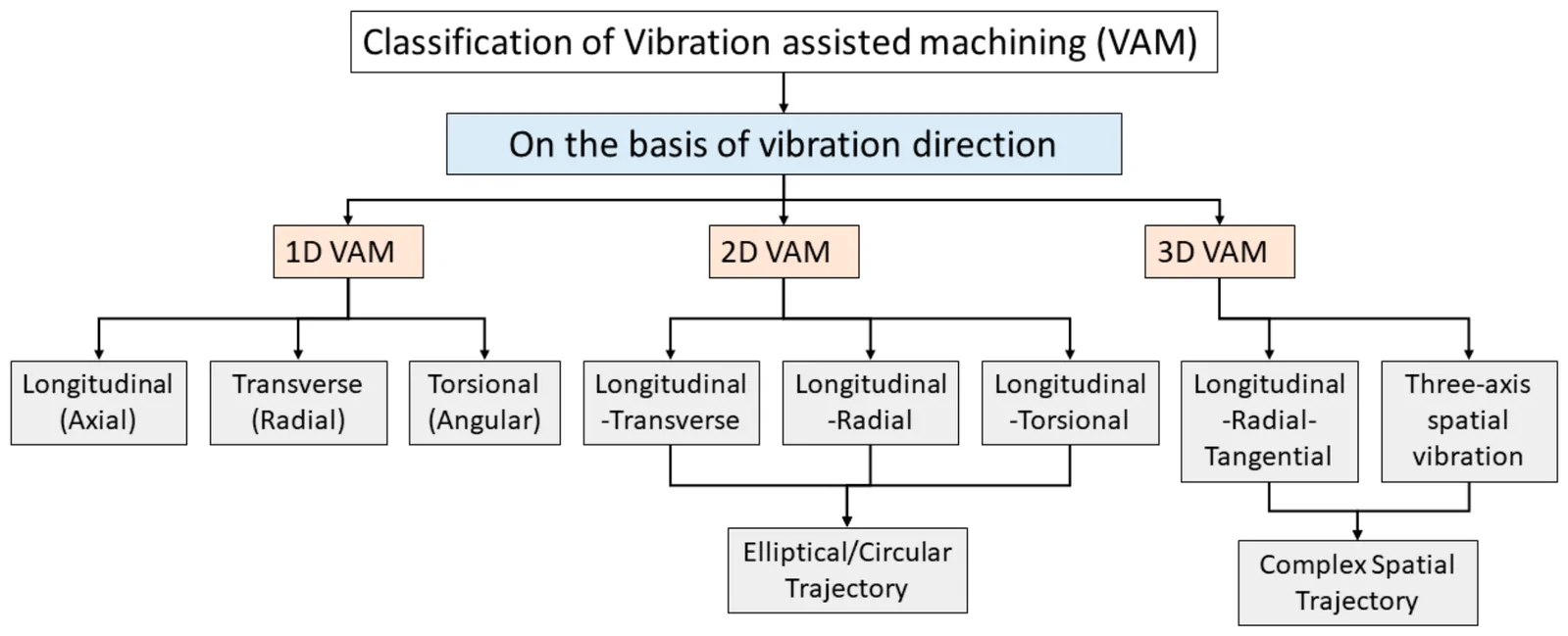
Classification of VAM process on the basis of vibration component application.

During one-dimensional (1D) VAM, vibration is applied along a single direction, usually the feed direction or depth-of-cut direction. This produces regular surface undulations, with their spacing determined by the relationship between the vibration frequency and the feed rate [33]. In two-dimensional (2D) VAM, vibrations are applied simultaneously along the two perpendicular directions, causing the tool tip to follow a Lissajous trajectory. A complete classification of VAM processes is illustrated in Figure 2. However, when the vibration amplitudes are equal and the phase difference is 90°, the tool tip traces a circular path, producing uniformly distributed isotropic dimple arrays [34]. Non-90° phase differences produce elliptical trajectories enabling anisotropic textures optimized for directional tribological or electrochemical functions [35].

Texture density—the ratio of textured to total surface area—is controlled jointly by depth of cut and feed per tooth. Typical texture densities for tribological applications range from 10% to 35% [36], while biosensor electrodes benefit from higher densities (40–50%) to maximize active surface area [37,38]. Importantly, the two application requirements place competing demands on the same geometric parameter, motivating the design of multifunctional hierarchical textures.

### 2.3. Surface Texture Morphologies Generated by VAM

**Figure 3:**
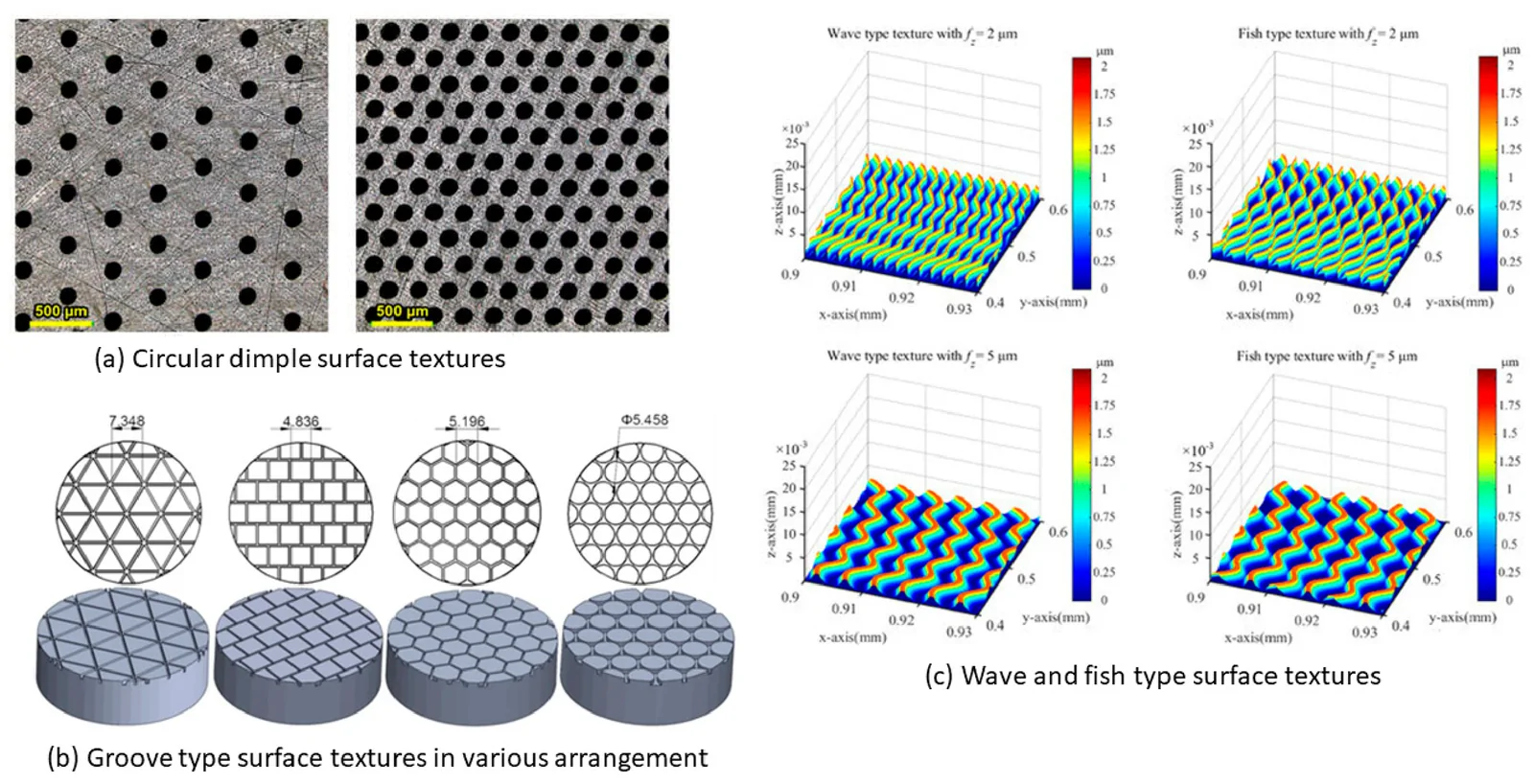
(**a**) Circular dimple [42], (**b**) groove [43], and (**c**) wave- and fish-type [16] surface textures (reprinted from Phun et al. [42], Hu et al. [43], and Song et al. [16] with permission from MDPI).

Vibration-assisted micro-milling can produce a wide variety of surface morphologies by only adjusting the machining parameters and vibration type. Two most common texture types recognized are (i) wave-type textures characterized by continuous sinusoidal undulations in the feed direction [9,39], and (ii) fish-scale textures in which each cutting edge pass creates a discrete scallop pattern overlapping with adjacent passes [4,40]. Fish-scale textures have shown excellent tribological performance due to their smooth, curved pockets which promote efficient hydrodynamic pressure generation while minimizing stress concentration around the texture edges. This characteristic gives them an advantage over the sharp-edged features commonly produced by laser ablation [41]. In addition to fish-scale textures, VAM can also produce a variety of other surface patterns, including dimple arrays, crosshatch grooves, pillar arrays, and hierarchical multi-scale textures using different machining strategies. Several studies have reported high geometric accuracy with relative errors of less than 1.8% for dimple depth and 0.7% for dimple length by using the VAM process [16]. Various surface texture morphologies are shown in Figure 3.

### 2.4. Process Parameters and Their Effect on Texture Quality

The quality of surface textures produced by VAM is mainly controlled by several machining parameters, including vibration frequency (f), vibration amplitude (A), spindle speed (n), feed per tooth (fz), axial depth of cut (ap), and tool geometry [25,44]. In general, the increase in vibration amplitude produces deeper textures with a higher aspect ratio, whereas increasing the feed per tooth increases the spacing between adjacent texture features. The ratio between spindle speed and vibration frequency also affects the periodicity of the surface textures along the feed direction [45,46]. Material properties, specifically the elastic modulus, yield strength, and thermal conductivity, play an important role by affecting the minimum uncut chip thickness (MUCT). When the uncut chip thickness falls below this critical value, elastic recovery becomes dominant and material removal no longer occurs [47]. Therefore, accurate modeling of elastic recovery is an important factor for predicting the final texture geometry, especially when machining soft metals and polymer substrates which are commonly used for flexible biosensor electrodes [48,49].

An important distinction should be made between the theoretical geometric resolution predicted from VAM kinematics and the practically manufacturable resolution obtained after material removal. The former is primarily governed by vibration amplitude and frequency, feed rate, cutting speed, tool trajectory, and tool geometry [45,46]. However, the final texture dimensions are also influenced by cutting edge radius, minimum uncut chip thickness (MUCT), elastic–plastic deformation, material properties, and elastic recovery [47,48,49]. When the instantaneous uncut chip thickness approaches or falls below the MUCT, stable chip formation may be replaced by plowing and elastic recovery, causing the actual texture geometry to deviate from the theoretically prescribed tool trajectory [47,48]. This limitation becomes particularly important for thin metallic films, coated surfaces, and compliant substrates, where substrate deformation and interfacial integrity can restrict the minimum reproducible feature size. Although the intermittent cutting mechanism of VAM can reduce cutting forces and burr formation under suitable conditions [50,51,52,53,54], excessive local deformation may still affect texture fidelity and surface integrity. Therefore, the practical resolution of VAM should be considered as a coupled process–tool–material–interface limitation, rather than solely as a function of vibration frequency and amplitude.

From a functional perspective, the VAM parameters should be selected according to the desired texture properties, rather than optimized separately. Texture depth and aspect ratio are mostly governed by the vibration amplitude, while texture repeatability, spacing and overlap depend on the vibration frequency and feed rate [45,46]. Cutting and spindle speeds influence the interaction between the tool motion and the vibration cycles, and hence the texture continuity and spatial distribution. Depth of cut and tool geometry influence the texture depth, width and edge profile [25,44]. These geometric characteristics directly affect the functional behavior of the textured surface. Texture depth and aspect ratio determine the capacity for lubricant or electrolyte retention, whereas texture spacing, density, and orientation influence lubricant distribution, debris entrapment, and fluid or analyte transport. Multidirectional vibration and multi-pass strategies can further be employed to generate crosshatched or hierarchical architectures with multiple functional characteristics [55,56,57]. Thus, a more rational design approach is to establish a clear process parameter → texture geometry → surface integrity → functional performance relationship, and then select the machining conditions according to the required tribological or electrochemical response. To further clarify these relationships, Table 1 summarizes the influence of the major VAM processing parameters on the resulting texture characteristics and their corresponding tribological and potential electrochemical functions, providing a direct link between process selection, texture design, and the targeted functional response.

## 3. Bio-Inspired Surface Texture Geometry and Design Principles

### 3.1. Classification of Surface Texture Geometries

Surface textures are classified according to their geometric dimensionality and spatial organization. Zero-dimensional (0D) surface textures consist of discrete closed-pocket features, mainly dimples and pits distributed across the surface [58]. One-dimensional (1D) surface textures generally include channels and grooves which are oriented parallel, perpendicular, or at an oblique angle to the main sliding or flow direction [59]. Two-dimensional (2D) surface textures include crosshatch patterns and waffle arrays while three-dimensional (3D) surface textures involve true topographic variation in all three spatial dimensions, which can be achieved through multi-pass VAM strategies [55,56,57]. However, the selection of a particular type of surface texture for a specific application requires consideration of the following main points: (i) the dominant lubrication regime; (ii) the dominant electrochemical mechanism; (iii) the biorecognition element chemistry; and (iv) the substrate material and operating environment [60,61].

### 3.2. Critical Geometric Parameters for Surface Textures

For both tribological and electrochemical applications, the functional performance of engineered surface textures is largely determined by their geometric design. Geometric parameters such as texture shape, depth, width, diameter, pitch, spacing, aspect ratio, texture density, and orientation govern key mechanisms including lubricant retention, contact stress distribution, wear debris entrapment, wettability, electrochemically active surface area, and reactant transport. Since these parameters directly affect frictional behavior, wear resistance, and electrochemical response, their careful selection and optimization are essential for achieving the desired performance in a specific tribological or biosensing application [60,62,63]. Some of the key surface texture parameters are discussed in more detail as follows:

- **Depth-to-diameter ratio (aspect ratio):** The depth-to-diameter ratio, commonly referred to as the aspect ratio, is an important geometric parameter as it determines the quantity of lubricant or electrolyte that can be retained within a surface texture. During tribological applications, shallow textures are unable to retain enough lubricant to provide effective lubrication, whereas overly deep textures may trap excessive wear debris and reduce the load-bearing capacity of the surface. As a result, many studies have reported that an aspect ratio between 0.1 and 0.3 provides a good balance between lubricant retention, hydrodynamic pressure generation, and friction reduction under mixed lubrication conditions [64,65].
- **Texture density (area coverage %):** Texture density, defined as the percentage of the surface covered by the surface textures, plays an important role in both tribological and electrochemical performance. In tribological applications, the load-bearing capacity of the surface and the amount of lubricant that can be retained within the texture are directly affected by the texture density. In case of electrochemical systems, an increase in texture density generally increases the electrochemically active surface area and improves reactant transport. However, if the texture density becomes too high, the structural support of the surface is reduced, which can cause the diffusion fields of neighboring features to overlap. Therefore, the texture densities in the range of 10–40% were generally considered to provide an optimum balance between tribological and electrochemical performance [66,67].
- **Edge sharpness:** The mechanical and electrochemical performance of textured surfaces depends strongly on the shape of texture edges. Sharp edges can create stress concentrations that promote crack initiation and localized wear during sliding motion. In electrochemical applications, sharp edges can also lead to uneven electric field and current density distributions across the electrode surface. In contrast, vibration-assisted machining (VAM) produces smoother edge transitions due to its intermittent cutting action. Hence, VAM textures can therefore reduce stress concentrations and improve the overall functionality of the textured surface [68,69].
- **Spatial arrangement:** The surface textures arrangement has a strong influence on the contact pressure, lubricant flow, and the transport of electrochemical species across the surface. Regular patterns, such as square and hexagonal arrays, generally result in more consistent tribological performance and predictable hydrodynamic behavior. On the other hand, random or bio-inspired texture arrangements lead to better replication of natural surfaces and have advantages in improving antifouling properties, cell adhesion, and biosensing performance. Hence, the selection of texture arrangement is primarily based on the specific functional requirements of the intended application [70,71,72].
- **Orientation relative to sliding/flow direction:** The surface texture orientation relative to the sliding or fluid flow direction plays an important role in both lubrication and mass transport phenomena. During tribological applications, surface textures aligned with the sliding direction generally promote lubricant entrainment and help reduce friction, whereas surface textures oriented perpendicular to the sliding direction are often more effective in trapping the wear debris. In case of electrochemical systems, texture orientation also affects the electrolyte flow, reactant diffusion, and charge transfer at the electrode surface. As a result, selecting an appropriate texture orientation is an important parameter for improving biosensor sensitivity and reducing response time [59,73,74].

**Figure 4:**
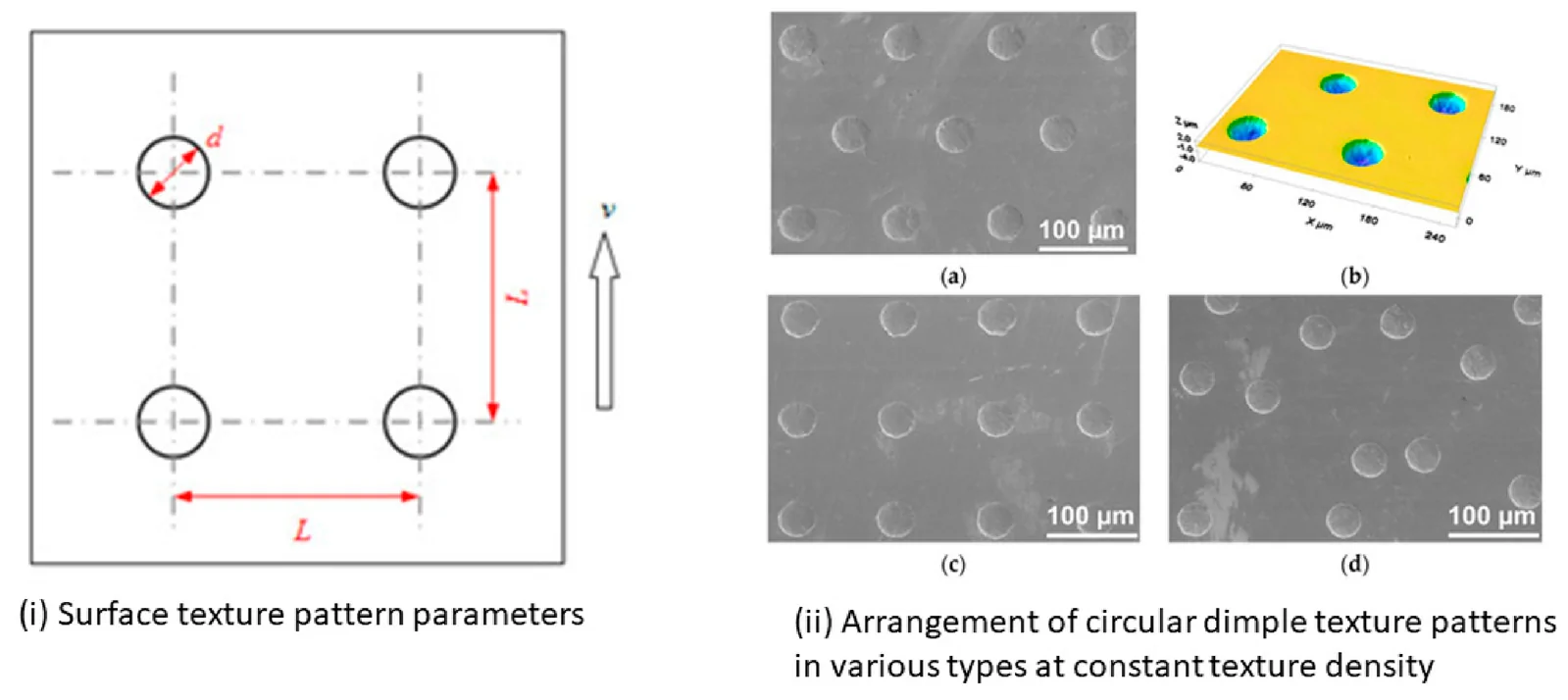
(**i**) Surface texture pattern parameters [75] and (**ii**) arrangement of texture pattern in different arrangement at constant texture density [76] (reprinted from Sun et al. [75] and Schneider et al. [76] with permission from MDPI).

The above discussion is summarized in Table 2, while various surface texture patterns and their geometric parameters are shown in Figure 4.

While the geometric parameters discussed above largely determine the functional performance of engineered surface textures, their successful implementation ultimately depends on the capabilities of the manufacturing process used to fabricate them. Implementation of different surface texturing techniques resulted in the generation of different types of surface textures, and their dimensional accuracy varies, with differential material compatibility, surface integrity, and suitability for large-scale manufacturing. Due to these factors, selection of the optimum texturing method is as important as optimizing the texture geometry to achieve the desired tribological or electrochemical performance. Table 3 compares the major surface texturing techniques used for tribological and biosensor electrode applications, highlighting their main advantages, limitations, and suitability for fabricating functional surface textures.

Among the available surface texturing techniques, vibration-assisted machining (VAM) has received increasing attention owing to its ability to produce high-precision textures while preserving the surface integrity of the substrate. Unlike thermal-based processes such as laser surface texturing (LST) and electrical discharge machining (EDM), VAM removes material through a mechanical cutting process with periodic tool–workpiece separation. Due to this, VAM application minimizes the formation of heat-affected zones, recast layers, thermal damage, and burr formation. Furthermore, the process inherently produces smoother texture edges and more accurate feature geometries, which are essential for maintaining uniform stress distribution in tribological contacts and homogeneous current density in electrochemical biosensor electrodes. Due to these inherent advantages VAM has become an attractive surface texturing technique in applications which require high geometric accuracy, enhanced surface integrity, and reliable functional surface performance.

However, these advantages should be considered together with the practical requirements of the manufacturing process. As summarized in Table 3, each texturing technique presents a different balance between equipment requirements, processing rate, material compatibility, surface integrity, reproducibility, and scalability. VAM requires a vibration generator, transducer–horn assembly, appropriate cutting tools, and accurate tool–workpiece alignment, while stable vibration conditions must be maintained throughout machining. Tool wear can also influence texture dimensions and long-term process reproducibility. Moreover, as texture generation generally follows the machining path, processing large surface areas may require longer fabrication times than high-speed laser scanning or batch-based lithographic techniques. LST, in comparison, provides flexible and non-contact pattern generation with good potential for large-area processing, although the equipment cost and possible thermal modification of the surface remain important considerations. Photolithography offers high repeatability and batch processing capability, particularly for planar substrates, but requires multiple processing steps and specialized facilities. Electrochemical and electrical discharge methods can also produce well-defined microfeatures, although their application is largely restricted to electrically conductive materials.

The technological value of VAM therefore lies not in replacing these established techniques, but in providing a complementary route where deterministic micro-scale geometry and preservation of surface integrity are important design requirements. This distinction becomes particularly relevant for hybrid micro–nanofabrication. For that, VAM can be used to establish the underlying micro-scale architecture, with controlled texture depth, spacing, orientation, and repeatability, while subsequent anodization, electrodeposition, hydrothermal treatment, thin-film deposition, or nanomaterial functionalization can introduce nano-scale and biochemical functionality. The two processing stages can therefore serve different purposes: the VAM-generated microstructure may regulate contact mechanics, fluid retention, directional transport, and mechanical stability, whereas the secondary treatment can increase electrochemically active surface area, improve charge transfer characteristics, or provide sites for bioreceptor immobilization.

Nevertheless, the additional manufacturing step must provide a measurable functional benefit to justify the increased process complexity. When sensing performance is governed mainly by nano-scale porosity, deposited functional materials, or surface chemistry, incorporating VAM may introduce additional tooling, alignment, processing time, and reproducibility requirements without providing a corresponding advantage. Therefore, the selection of a VAM-based hybrid route should be made on the basis of the required surface function and supported by comparison with the corresponding single-process surface. Such comparisons should consider not only sensing or tribological performance but also throughput, tool lifetime, reproducibility, material compatibility, and large-area scalability. This provides a more realistic basis for determining where VAM offers a genuine technological advantage in the fabrication of multifunctional and biomimetic sensing interfaces.

However, conventional single-scale surface textures are often designed for improvement of only one surface property, which limits their overall functionality. Consequently, increasing attention has been observed in the development of hierarchical surface textures which combine micro- and nano-scale features. The integration of surface textures at different length scales would result in improved lubrication, wear resistance, wettability, electrochemical activity, and enhanced overall surface performance.

### 3.3. Biological Inspiration and Translation into Engineered Textures

Natural surfaces provide useful design models for multifunctional interfaces because their performance frequently arises from organized features acting across several length scales. Rather than relying on a single geometric parameter, biological surfaces combine curvature, directional patterning, hierarchical roughness, and spatial gradients to regulate friction, adhesion, wetting, liquid transport, and fouling. For engineering translation, the key requirement is not the literal copying of biological appearance, but identification of the underlying structure–function relationship and its conversion into a geometry that can be manufactured reproducibly [5,77,78].

Fish-scale architectures provide a particularly relevant analogy for VAM as the overlapping curved units can be represented by the scalloped trajectories naturally generated during vibration-assisted milling. Recent biomimetic studies show that fish-scale patterns can alter interfacial lubricant transport and drag by controlling local flow paths and recirculation zones [4,79]. Similarly, shark-skin-inspired staggered grooves demonstrate how anisotropic micro-scale features can regulate near-wall flow and reduce shear losses [80]. For textured sensing interfaces, these principles can be translated into directional grooves, fish-scale patterns, or crosshatched features designed to guide electrolyte and analyte transport rather than merely increase roughness.

Other biological models emphasize interfacial rather than purely mechanical functions. Lotus leaves show how hierarchical micro-/nano-roughness can control wetting and self-cleaning [77], while gecko feet demonstrate the role of hierarchical fibrillar organization in controlling contact and adhesion [78]. Morpho butterfly wings further illustrate how multi-scale anisotropic topography can produce direction-dependent wetting behavior [4]. These examples are relevant to biosensor electrodes because wettability, receptor immobilization, biofouling, and mass transport are all sensitive to surface geometry. VAM can provide the deterministic micro-scale framework, whereas anodization, electrodeposition, hydrothermal growth, atomic layer deposition, or nanomaterial functionalization can subsequently introduce nano-scale features and biochemical specificity.

The resulting design route can therefore be expressed as biological model → functional principle → geometric abstraction → manufacturable VAM texture → interfacial function. This sequence is more rigorous than visual biomimicry alone as it allows each biological concept to be assessed against measurable engineering outputs such as friction coefficient, lubricant or electrolyte transport, contact angle, electrochemically active surface area, charge transfer resistance, antifouling behavior, and biosensor response. Table 4 summarizes representative biological inspirations and their possible translation into VAM-compatible functional textures.

**Figure 5:**
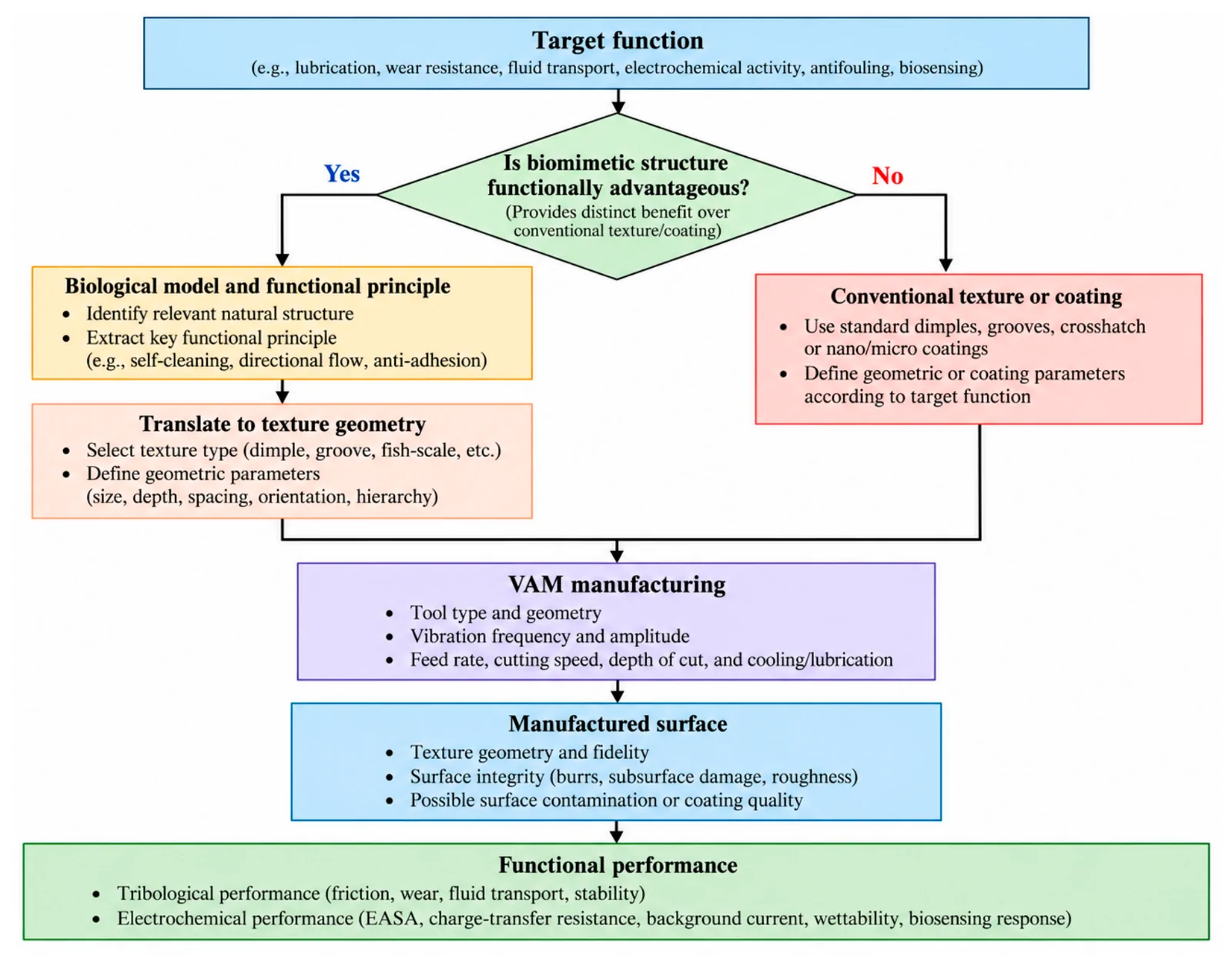
Function-driven framework for the design and manufacturing of VAM-generated biomimetic surface textures.

Based on these considerations, the selection of a biomimetic texture should begin with the required engineering function rather than with the morphology of the biological model. As summarized in Figure 5, the proposed framework follows a target function → biological principle → geometric abstraction → manufacturing parameters → surface characteristics → functional response pathway. The biological model is therefore introduced only when its hierarchy, directionality, curvature, or spatial organization provides a functional advantage that cannot be achieved as effectively by a simpler surface design. For example, conventional dimples or grooves may be sufficient when lubricant retention or contact-area reduction is the primary requirement, whereas fish-scale or directional architectures become more relevant when controlled fluid transport and contact regulation are required simultaneously. Similarly, nano-scale coatings may provide adequate electrochemical activity when surface chemistry is the dominant requirement, while hierarchical micro–nano-textures become useful when electrochemical functionality must be combined with fluid management, wettability control, or mechanical stability. Thus, biomimetic complexity should be justified by measurable functional gain rather than morphological resemblance alone.

### 3.4. Multi-Scale and Hierarchical Bio-Inspired Textures

Hierarchical organization is one of the most common design strategies found in biological surfaces. Natural interfaces rarely depend on a single characteristic length scale; instead, micro-scale structural elements are frequently combined with nano-scale roughness or surface chemistry to regulate several functions simultaneously [5,77]. Within engineered surfaces, these principles motivate the transition from conventional single-scale textures toward multi-scale and hierarchical architectures capable of combining tribological stability, wettability control, fluid transport, and electrochemical activity. Traditionally, micro-scale textures are extensively employed in tribological applications owing to their ability to enhance lubricant retention, reduce the actual area of contact, generate localized hydrodynamic pressure, and entrap wear debris. In contrast, nano-scale structures primarily modify physicochemical interactions at the solid–liquid interface by increasing roughness, wettability, electrochemically active surface area, and the density of biomolecule immobilization sites. Accordingly, a biomimetic hierarchical design should assign distinct but complementary functions to each structural scale rather than treating roughness simply as an additive surface feature.

**Figure 6:**
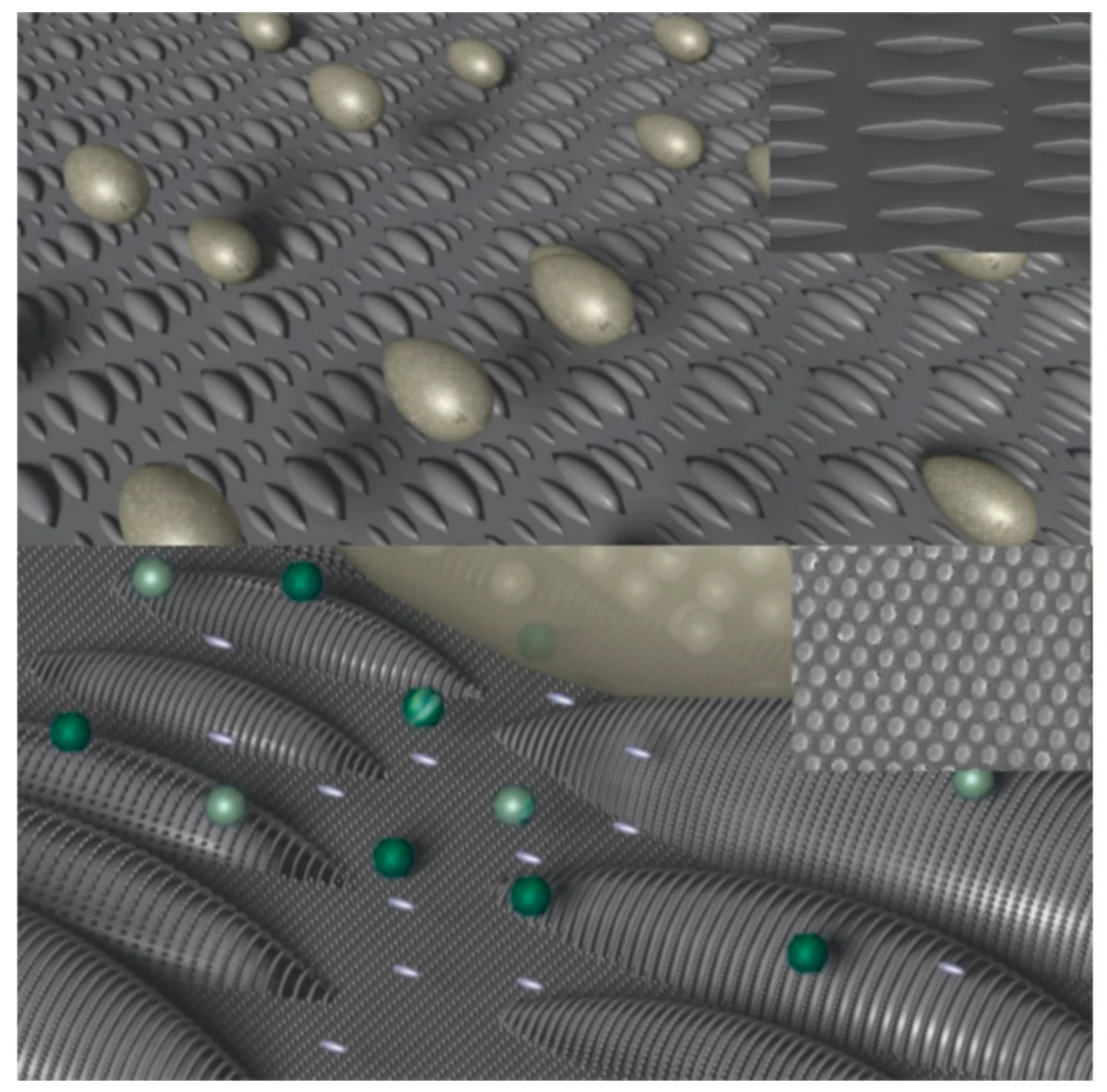
Metallic hierarchical structures with nanostructures on micro-riblets developed by Kim et al. [81].

Hierarchical surface textures are characterized by the intentional integration of structural features across multiple length scales, typically comprising deterministic micro-scale geometries superimposed with nano-scale roughness or functional nanostructures [61], as shown in Figure 6. Unlike conventional single-scale textures, hierarchical surface textures combine micro and nano-scale features to improve multiple surface characteristics simultaneously. The micro-scale surface textures mainly affect the contact mechanics by supporting the applied load, retaining lubricant, generating localized hydrodynamic pressure, and trapping the wear debris during sliding [66,67]. On the other hand, nano-scale textures improve the physicochemical properties of the surface by increasing the effective surface area, enhancing wettability, promoting electrolyte transport, and providing more active sites for biomolecule immobilization and electrochemical reactions [68]. The combination of these micro- and nano-scale surface textures results in improved friction and wear performance while also increasing mass transport and electrochemical activity. Due to these reasons, hierarchical surface textures often result in better overall performance in comparison to conventional single-scale texture designs.

From a tribological point of view, hierarchical surface textures offer numerous advantages in comparison to conventional single-scale micro-textures as they combine the benefits of both micro- and nano-scale features. Micro-scale textures improve lubrication by acting as lubricant reservoirs, generating localized hydrodynamic pressure, and trapping wear debris, which helps reduce direct contact between surface asperities during sliding [20]. Nano-scale surface textures further enhance the tribological performance by reducing the real area of contact, improving lubricant spread via capillary action, and aiding in maintaining the stable lubricant film under boundary and mixed lubrication environments. Nano-scale textures also modify the interfacial shear behavior and reduce adhesive interaction between the contacting surfaces, resulting in lower friction and improved wear resistance [82]. The combination of micro- and nano-scale surface textures at the same time results in more stable lubrication, adapting better to variable contact loads, and maintaining the optimum tribological performance over a wider range of operating conditions as compared to conventional single-scale textures [83].

In addition to the improvement in tribological performance, hierarchical surface textures can also enhance electrochemical and biosensing applications. Micro-scale surface textures facilitate efficient electrolyte circulation and mass transport, reducing concentration polarization near the electrode surface [84]. Simultaneously, nano-scale textures substantially increase the electrochemically active surface area, enhance charge transfer kinetics, and provide a greater density of adsorption sites for enzymes, antibodies, aptamers, and other biorecognition elements [85]. These improvements collectively increase sensor sensitivity, reduce detection limits, and enhance long-term operational stability. Moreover, the combination of micro- and nano-scale topographies allows precise control of surface wettability, which directly influences protein adsorption, cell attachment, and analyte diffusion parameters which are critical for implantable electrochemical biosensors [86,87]. However, these advantages will not necessarily be translated into an improved long-term biosensing performance. An increase in surface area, roughness, or nano-scale porosity can improve electrolyte transport and provide additional sites for bioreceptor immobilization, but the same features may also promote nonspecific adsorption of proteins and other biological species under physiological conditions. With prolonged exposure, the accumulation of these species within textured regions may partially block active sites and transport pathways, restrict analyte diffusion, alter surface wettability, and reduce the electrochemically accessible area. Therefore, hierarchical textures should be designed to achieve an appropriate balance between increased active area and efficient mass transport on one hand, and resistance to nonspecific adsorption and biological accumulation on the other. This consideration becomes particularly important for implantable and wearable biosensors, where continuous exposure to complex biological fluids can progressively modify the electrode–electrolyte interface and influence long-term sensing stability.

The fabrication of hierarchical surface textures requires manufacturing techniques which are capable of producing precise micro-scale textures while at the same time also preserving the surface integrity of the base material for subsequent nano-scale functionalization. Conventional thermal-based techniques, such as laser surface texturing (LST) and electrical discharge machining (EDM), are capable of producing complex surface geometries; however, they often result in undesirable thermal effects, including heat-affected zones (HAZs), recast layers, microcracks, and residual stresses, which may deteriorate the functional performance of the textured surface [88,89]. In contrast, vibration-assisted machining (VAM) has emerged as an effective mechanical surface texturing technique as it is successfully able to produce deterministic micro-scale textures with high dimensional accuracy and with minimal thermal damage [50,51]. The intermittent cutting mechanism, which is an inherent characteristic of VAM, results in reduced cutting forces, friction, and plastic deformation while producing smoother texture edges and fewer burrs [52,53,54]. These characteristics not only improve the quality of the fabricated micro-textures but also provide a suitable foundation for subsequent nano-scale functionalization using techniques such as electrochemical anodization, atomic layer deposition (ALD), and self-assembled monolayers (SAMs), enabling the fabrication of multifunctional hierarchical surfaces.

Following the fabrication of micro-scale surface textures by vibration-assisted machining (VAM), a number of nanofabrication techniques can also be employed to introduce secondary nano-scale features and construct hierarchical surface textures. Among these, electrochemical anodization has been widely used to produce nanoporous oxide layers on titanium alloys to enhance their surface wettability, corrosion resistance, and electrochemical activity [90,91]. On the other hand, atomic layer deposition (ALD) enables the deposition of uniform nanometer-thick functional coatings with excellent thickness control, whereas self-assembled monolayers (SAMs) facilitate molecular-level surface functionalization for selective biomolecule immobilization and improved interfacial interactions [92,93]. By combining these nano-scale modification techniques with the precise micro-textures produced by VAM, hierarchical surfaces with enhanced tribological and electrochemical performance can be fabricated while preserving the dimensional accuracy and surface integrity of the underlying micro-scale structures.

Recent studies clearly demonstrate the superior performance of hierarchical textures compared with conventional single-scale designs. For example, Seetharam et al. reported that hierarchical micro-/nano-textured Ti-6Al-4V surfaces exhibited approximately 35% lower friction coefficients together with nearly four-fold higher protein adsorption site density than comparable single-scale textured surfaces [94]. Huang et al. fabricated hierarchical micro-textured Ti-6Al-4V surfaces using longitudinal–torsional ultrasonic vibration-assisted milling (LTUVM). Compared with conventionally milled surfaces, the LTUVM-generated hierarchical textures exhibited enhanced surface hardness and hydrophilicity, and reduced friction coefficients and polymer wear rates under simulated oral lubrication, and promoted osteoblast adhesion and proliferation. These improvements are attributed to the synergistic effects of micro-pits acting as lubricant and wear debris reservoirs, together with enhanced oxide film formation, demonstrating the potential of VAM-fabricated hierarchical textures for simultaneously improving tribological performance and biocompatibility [95]. These findings demonstrate that integrating structural features across multiple length scales enables simultaneous enhancement of tribological performance and electrochemical functionality, making hierarchical textures particularly attractive for implantable biosensors, biomedical devices, and other multifunctional engineering systems. Consequently, the combination of deterministic micro-texturing through VAM with subsequent nano-scale surface functionalization represents one of the most promising strategies for developing next-generation multifunctional surfaces capable of simultaneously satisfying mechanical, tribological, and electrochemical requirements.

## 4. Tribological Functions of Bio-Inspired and VAM-Textured Surfaces

### 4.1. Mechanisms of Friction Reduction by Surface Texturing

The tribological benefits of engineered surface textures arise from several interacting mechanisms that collectively modify the contact conditions at the sliding interface. As compared to non-textured or smooth surfaces, textured surfaces change lubricant flow, contact pressure distribution, movement of wear debris, and interaction of surface asperities, which results in reduced friction and wear under a wide range of operating conditions [62]. The contribution of each type of mechanism to the improvement of tribological performance depends upon factors such as lubrication regime, texture geometry, and operating conditions. However, there are four mechanisms which were consistently recognized in the literature: hydrodynamic pressure generation, lubricant retention, wear debris entrapment, and reduction in the real contact area [96]. These mechanisms, instead of acting independently, work together to improve the overall tribological performance of textured surfaces.

One of the most common mechanisms responsible for friction reduction in textured surfaces is hydrodynamic pressure generation. When two surfaces slide against each other, surface textures such as micro-dimples, micro-grooves, or other designed textures change the way the lubricant flows through the contact area. These textures create small converging regions where the lubricant pressure increases, forming a thin lubricating film between the two surfaces. As a result, direct contact between surface asperities are reduced, leading to a lower friction and wear [64]. This mechanism is particularly effective under mixed and hydrodynamic lubrication conditions, where enough lubricant is available to form a stable film. The amount of hydrodynamic pressure generated depends on several factors, including the size, shape, depth, orientation, and density of the textures, as well as the sliding speed and lubricant properties. Many recent studies have shown that properly designed surface textures improve lubricant film stability and increase the load-carrying capacity of the contact, making hydrodynamic pressure generation one of the key reasons for the improved tribological performance of textured surfaces [97,98].

Another important mechanism is the lubricant reservoir effect. Surface textures could act as small lubricant storage sites which will trap the lubricant during operation and gradually release it into the contact area as the sliding continues. These micro-reservoirs help in maintaining a consistent lubricant supply while also avoiding lubricant starvation, especially during reciprocating motion, start–stop conditions, or interrupted sliding, where the lubricant film can easily become insufficient [99]. The stored lubricant supports the formation of a more stable lubricating film, which helps lower friction and reduce wear. Surface texturing using vibration-assisted machining (VAM) can further enhance the lubricant reservoir effect as the VAM process produces highly regular and uniformly distributed surface textures [95]. Surface textures such as dimples and fish-scale patterns generated by the intermittent cutting action of VAM retain lubricant more efficiently as compared to irregular surface features generated by conventional machining [100]. The regular texture patterns generated by VAM allow the lubricant to be distributed more uniformly throughout the sliding process, which results in more stable lubrication and improved tribological performance.

Entrapment of wear debris is another important mechanism by which surface textures improve tribological performance. During sliding motion, detached wear particles can become trapped between the sliding surfaces, where they act as third-body abrasives and can accelerate material removal and surface damage [73]. Optimum designed surface texture patterns can solve this problem by trapping the debris inside the micro-scale pockets before they re-enter the contact interface. Due to this, abrasive wear can be reduced, and the risk of severe scratching and surface damage is minimized. Debris entrapment becomes particularly important under boundary lubrication conditions, where direct contact between surface asperities is more common [101]. The ability to trap wear debris of textured surfaces depends upon factors such as texture size, volume, spacing, and distribution. In general, regular texture patterns are better than randomly rough surfaces as they provide well-defined and predictable debris storage sites [97].

In addition to improving lubrication, reduction in real area of contact is another important parameter by which surface textures enhance tribological performance. The introduction of micro-textures reduces part of the load-bearing surface area, due to which the actual area of contact between the opposing asperities is reduced under a given normal load [64]. As the adhesive friction primarily depends on the real contact area rather than the apparent contact area, this reduction in actual contact area helps to lower the adhesive component of friction [102]. Also, the discontinuous contact zone created by the surface textures also reduces the local stress concentrations and limits the growth of adhesive junctions between the opposing asperities, which aids in minimizing adhesive wear [100]. However, increasing the texture density beyond the optimum level can result in a reduction in the load-bearing capacity of the surface. This implies that an optimum texture density is needed to achieve the optimum balance between friction reduction and load support [103]. Therefore, careful selection of texture geometry remains an important parameter for improving tribological performance without compromising the structural integrity of the surface.

Recently, researchers have concluded that vibration-assisted machining (VAM) is an effective technique for producing high-quality surface textures with improved tribological performance. As compared to laser surface texturing (LST) and electrical discharge machining (EDM), VAM is a mechanical machining process which produces micro-textures without introducing heat-affected zones, recast layers, or significant thermal damage [64]. The intermittent cutting action, which is an inherent characteristic of the VAM process, also reduces cutting forces and plastic deformation, which produces surface textures with well-defined geometry, smooth edges, and good dimensional accuracy. Surface textures with these characteristics possess improved lubricant retention, reduced real area of contact, and enhanced wear debris entrapment, which ultimately leads to lower friction and wear. For example, Ali et al. reported that longitudinal ultrasonic vibration-assisted milling (LUVAM) produces uniform pattern surface textures which reduced the coefficient of friction and improved wear resistance under a starved lubrication environment [99]. Similarly, Huang et al. observed that hierarchical micro-textures fabricated on Ti-6Al-4V by longitudinal–torsional ultrasonic vibration-assisted milling (LTUVAM) exhibited lower friction and wear in comparison to conventionally machined surfaces while also promoting osteoblast adhesion and proliferation [95]. It has been concluded from these studies that VAM can produce precise and reproducible surface textures that will not only improve tribological performance but also provide additional functional advantages for applications such as biomedical implants.

### 4.2. Influence of Texture Parameters on Tribological Behavior

The tribological performance of textured surfaces depends not only on the presence of surface textures but also on their geometry, including texture shape, size, depth, area density, aspect ratio, orientation, and spatial arrangement [64]. These geometric parameters influence how effectively the textures generate hydrodynamic pressure, retain lubricant, trap wear debris, and reduce the real area of contact during sliding [100]. Over the past decade, a number of studies have focused on identifying the most suitable texture geometries for different lubrication regimes and loading conditions. However, recent reviews have shown that there is no single texture design which is suitable under all conditions. Instead, the optimum texture geometry depends on the combined effects of operating conditions, material properties, and fabrication method used [104].

The behavior of surface textures also changes significantly with the change in lubrication regime. Under hydrodynamic lubrication, where sliding surfaces are completely separated by a continuous lubricant film, texture geometry mainly influences lubricant retention, hydrodynamic pressure generation, and lubricant film stability, which together improve load-carrying capacity and reduce friction [105,106]. On the other hand, under boundary lubrication, direct contact between surface asperities becomes dominant. In this case, surface textures primarily act as lubricant reservoirs, trapping wear debris, reducing the real area of contact, and limiting adhesive interactions between the mating surfaces [107,108]. Due to these reasons, the most suitable texture design for a particular application depends upon the operating conditions, including sliding speed, applied load, lubricant viscosity, and contact pressure. Together, geometric parameters such as texture density, texture size, depth-to-diameter ratio, shape, orientation, and spatial arrangement all affect lubricant flow, pressure distribution, and wear debris transport [109]. Therefore, careful selection of these geometric parameters is essential for achieving low friction and high wear resistance under different lubrication environments.

Of all the different geometric parameters of surface textures, texture area density has been identified as one of the most important factors which influences friction and wear characteristics significantly. Texture area density is defined as the percentage of the surface covered by surface texture and controls the balance between lubricant storage and the available load-bearing area [72]. When the texture density is too low, surface texture pockets cannot retain enough lubricant, which results in limited hydrodynamic pressure generation and poor wear debris entrapment. On the other hand, an excessively high texture density reduces the effective load-bearing surface area and increases the stress concentration around the texture edges, which leads to an accelerated surface damage and reduced component life [72,110]. In recent studies it has been observed that texture area densities between 10% and 25% generally provided the best tribological performance under hydrodynamic lubrication as they generate sufficient hydrodynamic lift while maintaining adequate structural support [111]. On the other hand, under boundary and mixed lubrication conditions, slightly higher texture densities of 20–40% were often more effective, as the additional texture quantity improves lubricant retention and enhances wear debris entrapment during intermittent lubrication [20,112].

Surface texture depth is another important geometric parameter as it signifies how much lubricant would be stored within the texture and how effectively hydrodynamic pressure develops during sliding motion. Shallow textures are not able to retain enough lubricant and therefore contribute very little to hydrodynamic lubrication [113]. In comparison, excessively deep surface textures may promote lubricant recirculation and result in vortex formation inside the texture cavities, which results in reduced hydrodynamic pressure generation during sliding [114]. Due to this, the selection of an appropriate texture depth is important for a balance between lubricant storage and effective pressure generation. For circular-shaped dimples, both experimental and numerical studies have consistently shown that the optimum depth-to-diameter ratio lies between 0.10 and 0.30, although the exact value also depends on factors such as lubricant viscosity, sliding speed, and applied load [100]. Maintaining the aspect ratio within this range provides sufficient lubricant storage while minimizing pressure losses caused by internal flow separation.

Texture shape is another important surface texture characteristic which also significantly affects the tribological behavior of textured surfaces as surface textures with different geometries exhibit altered lubricant flow and stress distribution [100]. Circular dimples remain the most extensively investigated geometry owing to their simple fabrication and nearly isotropic hydrodynamic behavior [115]. However, elliptical dimples, grooves, chevron patterns, fish-scale textures, and biomimetic geometries have demonstrated their superior tribological performance under directional sliding conditions by promoting preferential lubricant transport and improving hydrodynamic pressure generation [116,117]. Fish-scale textures, in particular, have attracted considerable attention as in vibration-assisted milling the overlapping tool trajectories naturally generate deterministic fish-scale-like geometries which are capable of simultaneously improving lubricant retention and wear debris accommodation [4,118]. Recent reviews also suggest that asymmetric and multi-scale textures can outperform conventional circular dimples, although further optimization is required to understand the coupling effects between texture geometry and operating conditions [119,120,121].

In addition to texture geometry, the orientation and spacing of surface textures also play an important role in determining lubrication performance. For example, groove textures aligned parallel to the sliding direction generally show improved lubricant retention, whereas inclined or crosshatched grooves promote lubricant distribution in multiple directions and facilitate wear debris removal [122,123,124]. Similarly, texture spacing is equally important as it affects the interaction between the neighboring surface textures with one another. When surface textures are placed too close to one another, their pressure fields overlap, resulting in reduced hydrodynamic lift generation during sliding. On the contrary, if the spacing is too large, the textured regions become isolated and lubricant storage becomes limited, resulting in reduced overall effectiveness of the surface [125]. Due to these reasons, texture spacing was selected by considering both contact mechanics and lubricant flow on the basis of optimizing individual texture features independently. Recent studies have emphasized the importance of multi-parameter optimization, where several geometric parameters are optimized simultaneously to achieve the optimum tribological performance.

Recent advances in VAM processes has made it possible to produce textured surfaces with much better controlled geometric parameters. Unlike laser surface texturing (LST) and electrical discharge machining (EDM), VAM forms deterministic micro-textures through an intermittent cutting process without creating heat-affected zones, recast layers, or significant surface oxidation [39]. Hence, under appropriately controlled machining conditions, the periodic separation between the cutting tool and workpiece can reduce cutting forces and plastic deformation while producing consistent texture dimensions, smooth edges, and limited burr formation. Consequently, VAM enables more precise control over texture density, spacing, and feature geometry, leading to improved repeatability of tribological performance [126,127]. Furthermore, the excellent surface integrity produced by VAM provides an ideal substrate for subsequent nano-scale functionalization, enabling the fabrication of hierarchical surface architectures for multifunctional applications.

Overall, recent studies indicate that the tribological behavior of textured surfaces is effectively governed by the combined influence of multiple geometric parameters rather than by any individual texture parameter alone. Future research is therefore expected to focus on multi-objective optimization of texture geometry using numerical simulations, artificial intelligence, and data-driven design methods, particularly for deterministic textures fabricated by vibration-assisted machining, where high geometric precision allows the theoretical optimum texture design to be accurately reproduced in practical engineering applications. The approximate reduction in coefficient of friction with different surface texture patterns generated by VAM is summarized in Table 5.

### 4.3. Tribological Stability of VAM-Textured Surfaces in Biological Environments

Long-term tribological stability is a critical parameter for implantable and wearable biosensors, where metallic electrode surfaces are continuously exposed to complex physiological environments containing proteins, electrolytes, enzymes, and fluctuating mechanical loads [134]. Under these conditions, repeated sliding, micro-motion, and tribocorrosion can progressively degrade surface topography, leading to increased friction, accelerated wear, and deterioration of electrochemical sensing performance [135]. Hence, maintaining the integrity of engineered surface textures throughout long-term use is essential for ensuring stable sensor performance and reliable device operation. As compared with thermal surface texturing techniques, vibration-assisted machining (VAM) produces deterministic micro-textures through a mechanical material removal process, which avoids the formation of heat-affected zones, recast layers, and tensile residual stresses which can act as crack initiation sites during repeated loading [136]. As a result, VAM-generated textures exhibit high dimensional accuracy and a more uniform subsurface microstructure, providing a strong foundation for long-term tribological stability in biomedical applications [17].

Available studies suggest that the controlled geometry and surface integrity associated with VAM-generated textures can contribute to improved resistance against geometric degradation during repeated sliding [137]. The intermittent cutting action associated with VAM promotes localized plastic deformation and strain hardening beneath the textured surface while reducing machining-induced defects. As a result, surface textures such as micro-dimples, grooves, and fish-scale patterns are able to retain their geometrical structure more effectively during cyclic loading than laser-textured surfaces, where recast layers and thermally induced microcracks are more prone to wear-related spallation [102]. Also, the high dimensional accuracy of VAM-generated textures helps them to maintain lubricant retention, improve wear debris entrapment, and support stable lubricant film formation, allowing low friction to be sustained under boundary and mixed lubrication conditions. These advantages are particularly important for biosensor electrodes which experience continuous reciprocating motion and repeated mechanical loading during long-term implantation [138]. Although available studies provide encouraging evidence for the tribological performance of VAM-generated textures, long-term mechanical durability has been investigated less systematically than initial friction and wear response. Repeated sliding and cyclic loading can progressively round texture edges, reduce feature depth, or locally deform the textured regions, which may gradually change lubricant retention, debris entrapment, contact pressure distribution, and the effective load-bearing area. The extent of such degradation depends on the substrate material, texture geometry, applied load, sliding conditions, and surrounding medium. This distinction becomes particularly important for biomedical applications, where mechanical loading occurs together with protein adsorption, corrosion, and tribocorrosion. Therefore, the long-term advantage of VAM-generated textures should be established through repeated-cycle testing that monitors both texture geometry and functional response, rather than from the initial reduction in friction or wear alone.

Beyond mechanical durability, the long-term performance of VAM-textured surfaces is also dependent upon their resistance to tribocorrosion in biological environments. Stable surface textures reduce the real area of contact, distribute contact stresses more evenly, and help preserve the passive oxide film by minimizing localized stress concentrations. Together, these effects reduce the combined influence of mechanical wear and electrochemical corrosion [139,140]. In addition, as thermally induced defects are absent in VAM, it provides better coating adhesion and lowers the risk of localized corrosion around the texture edges. This property is particularly important for maintaining the long-term electrochemical performance of implantable biosensor electrodes [141,142,143]. Hence, it has been concluded that future studies should combine tribological testing, electrochemical impedance spectroscopy, and surface chemical analysis in both in vitro and in vivo environments in order to better understand how texture geometry, passive film stability, and sensing performance change over time. Combining VAM-generated hierarchical textures with biofunctional coatings and other surface engineering approaches could provide a practical route toward next-generation biosensor electrodes that maintain both tribological durability and electrochemical stability over long-term use.

### 4.4. Tribochemistry at Textured Biosensor Interfaces

Tribochemical reactions that occur at textured biosensor interfaces are of great importance for characterizing the tribological performance and long-term durability of implantable and wearable electrochemical devices [144]. Textured electrode surfaces are continuously exposed to biological fluids such as blood, interstitial fluid, and phosphate-buffered saline, in addition to mechanical contact which occurs alongside electrochemical reactions and biological adsorption. Unlike typical tribological systems which operate in oil-based lubricants, biosensor interfaces operate in complicated physiological environments which contain various types of proteins, lipids, amino acids, enzymes, and dissolved ions [145,146]. These biological components interact continuously with the textured surface, affecting passive oxide film formation, interfacial chemistry, and frictional behavior [147,148]. Furthermore, surface textures affect these processes by generating local variations in contact pressure, fluid flow, and the transport of the reactant within the individual surface texture. Due to these reasons, the tribochemical response, in addition to substrate material, also depends on texture geometry, feature size, spacing, and surface wettability [149,150,151]. Hence, understanding the complicated relationship between these mechanical, electrochemical, and biological processes is essential for designing textured biosensor electrodes which can maintain both long-term mechanical durability and reliable sensing performance.

Surface texturing offers a number of tribochemical benefits which are useful in extending the service life of biosensor electrodes. Micro-scale textures help in retaining physiological fluids which are gradually released back into the contact interface during sliding which improves lubrication, reduces direct contact between the asperities, and results in minimized adhesive wear [152,153]. Also, surface texturing aids in trapping wear debris, which prevents the abrasive particles from continuously passing through the contact zone and leads to reduced friction and wear. Apart from that, proteins adsorbed within textured regions form hydrated boundary films which provide additional lubrication under low-load conditions [154,155]. Despite the above-mentioned advantages, there are some challenges which remain unsolved for surface-textured electrodes. For example, an excessive protein adsorption can result in biofouling, which limits analyte transport to the electrochemically active surface and leads to reduced sensor sensitivity [156,157]. This distinction is particularly important because a texture showing high EASA and rapid charge transfer in a simple model electrolyte may respond differently after exposure to protein-rich physiological media. Adsorbed proteins and other biological species can progressively block active sites and transport pathways, causing changes in interfacial impedance, background current, wettability, and sensor response even when the original texture geometry remains unchanged. In the same way, too deep or poorly designed surface textures can result in the accumulation of chloride ions and biological residues, which increases the risk of passive film breakdown, localized corrosion initiation, and tribocorrosion [158,159]. In addition, continuous repeated mechanical loading can also change the geometry of the surface textures, which affects both lubricant retention capacity and electrochemical performance. Due to these reasons, texture dimensions should be carefully selected in order to achieve an optimum balance between improved tribological performance and sufficient electrochemical accessibility for reliable long-term sensing ability.

The tribochemical behavior of textured biosensor surfaces is strongly affected by texture geometry, with different surface texture patterns affecting lubrication performance, contact mechanics, and the interaction with the biological environment through different methods [160,161]. For example, micro-dimpled textured surfaces mainly act as lubricant reservoirs and as traps for wear debris, which helps in maintaining stable friction and reducing wear under lubricated conditions [162]. On the contrary, linear groove-type texture patterns guide the movement of biological fluids and reaction products along the preferred direction; however, their frictional behavior can vary with the sliding direction [163]. Crosshatched surface textures provide interconnected pathways for lubricant flow, which improves fluid replenishment and distributes contact stresses more evenly, which makes them specifically beneficial under reciprocating sliding conditions [122]. Also, hierarchical micro-/nano-surface textures, including those produced by VAM, combine lubricant storage with capillary effects and improve fluid retention, surface wettability, and passive oxide film stability [164]. Similarly, biomimetic fish-scale surface texture patterns guide the fluid flow and redistribute the contact stresses, whereas pillar-array-type texture patterns reduce the real area of contact and improve load distribution. However, densely packed pillars can promote localized protein accumulation [165,166].

It is concluded that future research should focus on establishing a clear quantitative relationship between texture geometry, tribochemical reactions, passive film behavior, protein adsorption, and electrochemical sensing ability. Understanding of such a complicated relationship would provide a stronger foundation for designing a multifunctional biosensor electrode with predictable performance [167,168]. In recent studies it has also been observed that controlling tribocorrosion, protein–surface interactions, and oxide film stability is very important for maintaining the long-term performance of metallic biomedical appliances/surfaces and functional coatings in physiological environments [169,170]. Due to these reasons, combining VAM-generated surface textures with suitable tribochemical properties could result in improved wear resistance, corrosion stability, and resistance to biofouling while maintaining a stable electrochemical response during long-term operation.

## 5. Interfacial and Electrochemical Functions of Textured Surfaces

The electrochemical effects discussed in this section should be considered according to the available level of experimental evidence. While the ability of surface texture geometry to influence electrochemically active area, mass transport, diffusion behavior, and charge transfer kinetics is well established for structured and nanostructured electrodes, direct experimental validation of these mechanisms on VAM-fabricated biosensor electrodes remains limited. Therefore, where VAM-specific evidence is not available, the reported mechanisms are discussed as structure–function principles that may be translated to VAM-generated surfaces rather than as experimentally demonstrated VAM effects. Within this framework, VAM primarily provides deterministic control of the micro-scale texture geometry, whereas subsequent nano-scale modification can introduce additional electrochemical activity, surface chemistry, and biomolecular functionality.

Despite the tribological and tribochemical benefits of VAM surface textures which offer a mechanical basis for improving the longevity and dependability of biosensor electrodes, the overall efficiency of these devices primarily depends upon the electrochemical reactions at the electrode–electrolyte interface. Surface texturing alters frictional dynamics and wear progression while significantly affecting electron transfer, electrochemically active surface area (EASA), analyte movement, interfacial wettability, and biomolecular adhesion, which collectively controls the sensitivity, selectivity, response time, and operational stability of electrochemical biosensors. Compared to traditional flat electrodes, textured surfaces have regulated three-dimensional topographies which produce localized discrepancies in electric field distribution, diffusion routes, and reaction kinetics, which enhances electrochemical performance and outweighs the typical material selection of [171,172,173]. Additionally, the deterministic micro and hierarchical textures generated by VAM results in enhanced dimensional precision, reduced thermal damage, and outstanding surface integrity, enabling meticulous regulation of these interfacial phenomena while maintaining mechanical durability, which is necessary for extended use in biological settings. Recent developments in microfabrication and surface engineering have consequently transformed the design approach of biosensor electrodes from simply enhancing surface roughness to developing multifunctional surface architectures which can concurrently improve tribological resilience and electrochemical performance [174,175]. Due to this, understanding the impact of VAM-produced surface textures on electrochemical performance has emerged as a vital research focus at the convergence of precision manufacturing, tribology, electrochemistry, and biomedical engineering. The effect of surface texture patterns on electrochemically active surface area, electron transfer, mass transport, electrochemical stability, and biofunctionalization is discussed in the following subsections. These aspects are examined in order to better understand the effect of surface texture on sensing performance and to identify suitable design approaches for developing textured biosensor electrodes.

### 5.1. Electrochemically Active Surface Area

The electrochemically active surface area (EASA) is one of the most influential parameters governing the analytical performance of electrochemical biosensors as it directly determines the number of accessible reaction sites available for heterogeneous electron transfer [176,177]. Unlike the geometric surface area, EASA represents the fraction of electrode surface that actively participates in electrochemical reactions and is commonly determined using cyclic voltammetry (CV) with the ferri/ferrocyanide ([Fe(CN)_6]^{3-/4-}) redox couple through the Randles–Ševčík relationship [178]. A larger EASA generally leads to more active sites for electrochemical reactions, resulting in higher Faradaic current, better signal-to-noise ratio, improved sensitivity, and lower detection limits. Due to this, EASA is commonly regarded as an important parameter of electrochemical performance in case of textured electrodes [179,180]. Recent studies have also shown that micro-textured electrodes fabricated using different surface texturing techniques have EASAs approximately 2–8 times greater as compared to geometrically equivalent planar electrodes. However, the increase in EASA depends mainly on texture density, texture depth, aspect ratio, and the distribution of surface textures across the electrode surface [100,181,182].

From a VAM perspective, comparable geometric mechanisms may be expected where deterministic micro-textures increase the real surface area and modify local mass transport; however, direct electrochemical validation of these effects on VAM-fabricated biosensor electrodes remains limited. Micro-dimples, micro-pockets, and hierarchical surface features substantially increase the real surface area while simultaneously introducing numerous edge and corner sites that possess higher local electrochemical activity than flat regions [71]. These geometrical discontinuities promote radial or hemispherical diffusion of electroactive species around texture boundaries, analogous to the diffusion behavior observed in ultramicroelectrode arrays, which is superimposed on the conventional planar diffusion field [183,184]. Consequently, analyte replenishment at the electrode interface is accelerated, resulting in higher steady-state current densities and improved electrochemical response [185]. Furthermore, the deterministic and thermally damage-free nature of the VAM methodology minimizes surface defects, recast layers, and oxide heterogeneity, resulting in preserved intrinsic conductivity of the substrate and ensuring uniform electrochemical activity across the textured surface [186]. Recent studies have also indicated that optimizing texture geometry is essential as excessively high texture densities may lead to overlapping of diffusion layers, which reduces the effective utilization of active sites despite an increase in geometric area [74,187]. Therefore, achieving an optimal balance between texture dimensions, spacing, and surface coverage is critical for maximizing EASA and enhancing the overall sensing performance of biosensor electrodes textured using VAM.

### 5.2. Mass Transport Enhancement

Mass transport is a key factor in controlling the performance of electrochemical biosensors as it determines how rapidly analyte molecules are delivered from the bulk solution to the electrode surface, where electrochemical reactions occur [188,189]. The overall response of all the sensing systems is governed not only by the intrinsic electron transfer kinetics of the electrode material but also by the rate at which target molecules diffuse through the electrolyte [172,190]. Consequently, the improvement in mass transport is an effective strategy for increasing current response, shortening response time, and lowering the detection limit [191,192]. Surface texturing provides an effective method for modifying the local flow dynamics without changing the electrode’s chemical composition [176,177]. In comparison to conventional planar electrodes, textured surfaces create three-dimensional micro-textures that modify concentration gradients and diffusion pathways, thereby enhancing the transport of electroactive species to the active surface [164,193].

In case of stagnant electrolytes where molecular diffusion is the major transport mechanism, surface textures such as micro-dimples, grooves and textured pockets can operate as concentrated analyte reservoirs [193,194,195]. These surface textures temporarily retain electrolytes in the textured area and increase the retention time of analyte molecules in the vicinity of the electrode surface and reduce diffusion distances during electrochemical reactions [196,197,198]. Therefore, the local concentration of electroactive species is higher in textured electrodes in comparison to smooth electrodes, resulting in better current stability and enhanced sensing performance. Besides that, the edges of surface textures facilitate multidirectional diffusion, enabling additional analyte molecules to access the reaction sites more effectively compared to on planar surfaces, where diffusion is predominantly one-dimensional [199,200]. However, the beneficial effects depend primarily on texture geometry, as excessively deep or densely packed textures may impede electrolyte renewal and create diffusion overlap between neighboring features, leading to a reduced transport efficiency [74,201].

Apart from that, under dynamic flow conditions, such as those encountered in microfluidic biosensors and implantable sensing devices, the influence of surface texture becomes even more pronounced. The interaction between the flowing electrolyte and engineered microstructures generates localized recirculation zones and micro-vortices that continuously disturb the diffusion boundary layer adjacent to the electrode [118,202]. This thinning of the diffusion layer accelerates analyte replenishment and increases mass transfer rates, resulting in higher amperometric currents and faster sensor responses [203,204]. These mechanisms have been demonstrated mainly for structured electrodes and through computational or microfluidic studies, and should not yet be interpreted as direct experimental evidence for VAM-fabricated biosensor electrodes. Nevertheless, VAM offers deterministic control over micro-scale features such as texture depth, spacing, orientation, and periodicity, which are parameters known to influence local fluid transport. Fish-scale, groove, and hierarchical micro-textures produced by VAM may therefore provide a controllable platform for regulating electrolyte transport and reducing stagnant regions; however, direct electrochemical and flow-field validation is still required to quantify these effects specifically for VAM-textured biosensor surfaces [118,203]. Consequently, VAM-fabricated textured electrodes offer significant potential for enhancing the sensitivity, response speed, and long-term operational stability of electrochemical biosensors, particularly during continuous monitoring applications where efficient mass transport is essential [188,189,205].

### 5.3. Charge Transfer Kinetics on Textured Surfaces

Charge transfer kinetics strongly influence the performance of electrochemical biosensors as they determine how efficiently the electrons are exchanged between the electrode surface and electroactive species in the electrolyte. Electrochemical impedance spectroscopy (EIS) is one of the most commonly used techniques to analyze this phenomenon, with the charge transfer resistance (Rct) indicating the facilitation of electron movement across the electrode–electrolyte interface. A lower Rct generally indicates faster electron transfer and improved electrochemical performance [206,207]. Recent studies have also shown that micro- and nano-textured electrode surfaces exhibit significantly lower charge transfer resistance than smooth electrodes as the textured electrode surface provides a larger electrochemically active surface area and a greater number of accessible reaction sites [208,209,210,211]. These characteristics facilitate electron exchange, resulting in higher current responses and improved sensing performance.

In addition to increasing the active surface area, textured electrodes modify the local interfacial electrochemical environment. The larger real surface area reduces the local current density for a given applied current, which decreases the contribution of double-layer charging relative to the Faradaic current [212,213,214]. Consequently, a greater proportion of the measured signal originates from the target electrochemical reaction, leading to improved signal-to-background ratios and higher detection sensitivity [207,215]. Furthermore, the periodic mechanical deformation associated with vibration-assisted machining (VAM) can expose fresh metallic surfaces, refine the near-surface microstructure, and introduce crystallographic defects such as dislocations and grain boundaries [216,217]. These microstructural changes create additional electrochemically active sites and shorten the electron transfer pathway, thereby increasing the heterogeneous electron transfer rate constant (k°). As a result, VAM-textured electrodes show considerable promise for improving the sensitivity, repeatability, and long-term stability of electrochemical biosensors [218,219,220].

### 5.4. Wettability, Biofunctionalization, and Antifouling

Surface wettability is one of the most important interfacial properties influencing the electrochemical performance of textured biosensor electrodes as it governs electrolyte spreading, biomolecule immobilization, and the interaction between the sensing surface and biological media. Wettability is commonly characterized by the water contact angle (WCA), where hydrophilic surfaces generally facilitate electrolyte penetration and improve contact between the electrode and the surrounding solution, while hydrophobic surfaces restrict liquid spreading and reduce effective interfacial contact [221,222,223]. Surface texturing produced by vibration-assisted machining (VAM) provides a practical approach for tailoring wettability by controlling texture geometry, roughness, and surface energy. Depending on the texture dimensions and intrinsic surface chemistry, the wetting behavior can be explained using the classical Wenzel and Cassie–Baxter models, allowing the surface to be engineered from hydrophobic to highly hydrophilic states [224,225].

The wettability of textured electrodes plays a crucial role in biofunctionalization. Hydrophilic textured surfaces (typically WCA < 60°) promote uniform electrolyte infiltration and provide favorable conditions for the immobilization of thiol-based self-assembled monolayers (SAMs), antibodies, enzymes, and other biorecognition molecules on metallic electrodes [145,225,226]. Improved molecular coverage enhances the availability of active binding sites, resulting in more reproducible biosensor responses and higher analytical sensitivity [145,227]. In addition, the deterministic microstructures generated by VAM offer a larger effective surface area, enabling more homogeneous distribution of immobilized biomolecules while minimizing localized defects that may adversely affect sensing performance.

Another important advantage of textured electrode surfaces is their compatibility with antifouling surface modifications. Studies have shown that combining textured substrates with hydrophilic polymer coatings, particularly zwitterionic polymers and polyethylene glycol (PEG), significantly suppresses nonspecific protein adsorption and improves sensor performance in complex biological fluids [223,226,227,228]. These coatings form stable hydration layers that act as physical and energetic barriers against protein attachment, while the curvature and topographical variation introduced by surface texturing further disrupt the formation of continuous protein films [228,229]. Consequently, the synergistic combination of engineered surface textures and antifouling coatings provides improved resistance to biofouling, enhances signal stability, and extends the operational lifetime of electrochemical biosensors intended for continuous biological monitoring [145,226].

## 6. Translation of Bio-Inspired Textured Interfaces to Electrochemical Biosensing

The electrochemical characteristics of textured electrode surfaces discussed in the preceding sections provide an important basis for their translation to different biosensing applications. However, it is important to distinguish between functional improvements that have been demonstrated directly on VAM-fabricated surfaces and those reported for structured or nanostructured electrodes produced using other fabrication techniques. At present, direct experimental studies employing VAM specifically for electrochemical biosensor fabrication remain limited. Therefore, several biosensing examples discussed in this section were obtained using laser processing, electrodeposition, hydrothermal synthesis, lithography, nanomaterial deposition, or other surface-modification approaches. These studies are included to establish relevant structure–function relationships, particularly the influence of surface architecture on electrochemically active area, electron transfer, mass transport, biomolecule immobilization, and interfacial wettability, and should not be interpreted as direct experimental evidence of VAM-enabled biosensing. Where VAM has been used experimentally, this is identified explicitly. For studies based on other fabrication routes, their relevance to VAM is discussed only from a translational perspective, considering whether comparable geometric or hierarchical features could potentially be produced through VAM or hybrid VAM-based surface functionalization.

### 6.1. Metabolic Biomarker Detection

#### 6.1.1. Glucose and Hydrogen Peroxide Detection

Glucose detection is one of the most widely investigated applications of electrochemical biosensors owing to its indispensable role in diabetes diagnosis and continuous metabolic monitoring [230,231,232]. The sensing performance of both enzymatic and non-enzymatic glucose biosensors is largely determined by the physicochemical characteristics of the electrode interface, where an increase in electrochemically active surface area, enhanced electron transfer kinetics, and efficient mass transport collectively contribute to improved analytical performance [233,234]. Consequently, micro- and nano-textured electrode surfaces have attracted considerable attention as they provide a greater density of accessible catalytic sites, facilitate analyte diffusion, and increase the effective loading of catalytic materials or biorecognition elements [235,236]. These characteristics generally result in higher sensitivity, shorter response times, lower detection limits, and improved operational stability compared with conventional planar electrodes [237,238]. In enzymatic biosensors, textured architectures promote the uniform immobilization and retention of glucose oxidase (GOx), whereas non-enzymatic sensors employing nickel-, copper-, platinum-, and transition metal oxide-based electrodes benefit from the increased availability of electrocatalytically active sites for glucose oxidation [234,237,238]. Likewise, hydrogen peroxide, which serves both as an important biomarker of oxidative stress and the reaction product of numerous oxidase-based biosensors, can be detected with high sensitivity using hierarchically textured catalytic electrodes due to their rapid charge transfer and enhanced mass transport [239,240]. Although the application of vibration-assisted machining (VAM) for fabricating electrochemical biosensor electrodes is still at an early stage, its capability to produce deterministic micro and hierarchical surface textures with high dimensional accuracy [20,241] provides a promising manufacturing strategy for the next generation of high-performance glucose and hydrogen peroxide biosensors.

#### 6.1.2. Urea and Other Small Metabolites

Urea is a clinically important metabolic biomarker widely used for evaluating renal function, monitoring chronic kidney disease progression, and assessing the effectiveness of hemodialysis treatment. Recently, continuous urea monitoring has attracted increasing interest for wearable and point-of-care healthcare systems as it enables real-time assessment of physiological status [242,243]. The performance of electrochemical urea biosensors is largely governed by the physicochemical characteristics of the electrode surface. Engineered micro- and nano-textured electrodes provide an increased electrochemically active surface area, improve urease immobilization, and facilitate electron transfer across the electrode–electrolyte interface, thereby enhancing catalytic efficiency and analytical performance [242,244]. These structural characteristics generally, as also mentioned earlier, lead to higher sensitivity, lower detection limits, faster response times, and improved operational stability compared with conventional planar electrodes [245]. Similar electrode engineering strategies can also be extended to the electrochemical detection of other clinically relevant metabolites, including creatinine, lactate, and uric acid, where textured surfaces could improve biomolecule immobilization, promote efficient mass transport, and increase the availability of electrochemically active sites [144,243]. Although the application of VAM for manufacturing metabolite biosensor electrodes is still in its infancy, the capability of VAM to produce deterministic micro and hierarchical surface textures with excellent dimensional accuracy and surface integrity [246] presents a promising opportunity for developing next-generation wearable and implantable electrochemical sensing platforms.

### 6.2. Cancer Biomarker Detection

Early diagnosis of cancer increasingly depends on the accurate detection of protein biomarkers, including carcinoembryonic antigen (CEA), prostate-specific antigen (PSA), alpha-fetoprotein (AFP), and various cytokines, whose concentrations in biological fluids are often present within the range of pg/mL or fg/mL [247,248]. Consequently, electrochemical immunosensors require electrode interfaces which provide high sensitivity, rapid electron transfer, and excellent antibody immobilization. Engineered micro- and nano-textured electrodes result in an increased electrochemically active surface area, improving charge transfer kinetics, and providing a greater density of accessible binding sites for antibodies and aptamers which satisfy these requirements substantially [248,249]. Gold-based electrodes functionalized with thiol self-assembled monolayers (SAMs) remain the most widely used platform as they enable stable and highly oriented immobilization of biorecognition molecules, while hierarchically structured interfaces further enhance biomolecule loading and electrolyte accessibility [250,251,252,253]. In addition, label-free electrochemical impedance spectroscopy (EIS) has become an effective strategy for cancer biomarker detection, where antigen–antibody interactions produce measurable changes in charge transfer resistance (Rct), enabling highly sensitive and selective analysis of multiple cancer biomarkers [248,254,255,256,257].

### 6.3. Nucleic Acid and Genetic Biomarker Detection

Electrochemical detection of nucleic acids, including microRNAs (miRNAs), circulating cell-free DNA (cfDNA), and viral RNA, has gained considerable attention for early disease diagnosis, liquid biopsy, and rapid identification of infectious diseases [258,259,260]. On the contrary, due to the extremely low quantity of these biomarkers in biological samples, electrode interfaces capable of performing efficient probe immobilization, quick target hybridization, and good signal amplification are required [261,262]. Micro- and nano-textured electrodes offers distinct benefits in this respect such as increasing the electrochemically active surface area and providing a greater number of accessible sites for immobilizing DNA probes, aptamers, and other recognition molecules [260,263,264,265]. Integration of textured surfaces with two-dimensional materials, including graphene and MoS_2_, can further improve electrical conductivity and facilitate interfacial electron transfer [265,266,267], while amplification strategies such as hybridization chain reaction (HCR) considerably enhance the electrochemical response at very low target concentrations [268,269]. Moreover, the three-dimensional organization of recognition probes and textured electrode interfaces can improve target accessibility and hybridization efficiency, thereby increasing the sensitivity and reliability of nucleic acid detection [262,270]. These characteristics make precisely textured electrode topographies particularly attractive for highly sensitive and potentially multiplexed genetic biomarker detection.

### 6.4. Neurotransmitter and Neurochemical Sensing

Electrochemical monitoring of neurotransmitters such as dopamine (DA), serotonin (5-HT), and norepinephrine (NE) is important for understanding neuronal signaling and the progression of neurological and psychiatric disorders. However, detection of these neurotransmitters in biological environments is challenging due to their low concentrations and the presence of electroactive interferents, particularly ascorbic acid (AA) and uric acid, which can produce overlapping oxidation responses [271,272,273]. Hence, over recent years, a number of studies have focused focused on micro- and nanostructured carbon-based electrodes, in which increased electrochemically active surface area, improved conductivity, and abundant accessible reaction sites enhance neurotransmitter oxidation and improve analytical sensitivity [274,275].

CNT, graphene, and nanographitic carbon-modified interfaces have demonstrated improved selectivity for dopamine and serotonin, particularly for simultaneous detection in complex biological environments [272,276,277]. Boron-doped diamond (BDD) electrodes are also gaining attention for neurochemical sensing because of their wide electrochemical potential window, low background current, chemical stability, and resistance to surface fouling [278,279]. These characteristics are particularly important for continuous measurements, where electrode passivation and signal drift can compromise long-term sensing performance. Consequently, engineered electrode architectures provide a promising platform for sensitive, selective, and potentially multiplexed monitoring of neurochemical dynamics.

### 6.5. Wearable and Point-of-Care Biosensors

The integration of electrochemical biosensors into wearable platforms, including epidermal patches, smart textiles, and microneedle arrays, requires electrodes that combine mechanical flexibility, conformability, and stable electrochemical performance during repeated body motion [144,280,281]. Recent developments in flexible nanostructured electrodes and conductive polymer composites have improved interfacial contact while maintaining electrical and sensing characteristics under mechanical deformation [282,283]. Such architectures are increasingly being employed for continuous monitoring of metabolites, electrolytes, and stress-related biomarkers in sweat and interstitial fluid [144,280,284].

Cortisol sensing is particularly attractive for wearable systems, with recent aptamer, hydrogel, and nanomaterial-based electrochemical platforms enabling sensitive measurements directly from sweat [285,286]. Beyond electrochemical performance, the mechanical and tribological characteristics of the skin–sensor interface are equally important for reliable on-body operation. Improved conformability and stable adhesion minimize relative movement at the interface, thereby reducing motion-induced signal artifacts and maintaining consistent sensor–skin contact during prolonged wear [287,288,289]. These considerations make controlled surface architecture (surface textures) specifically relevant when designing mechanically robust and comfortable wearable biosensing interfaces.

### 6.6. Environmental and Food Safety Monitoring

Electrochemical monitoring of toxic heavy-metal ions, particularly Pb^2+^, Cd^2+^, Hg^2+^, and As^3+^, is important for assessing contamination in water and food systems. It has been observed that porous and nanostructured bismuth/carbon electrodes are particularly effective for stripping-voltammetric analysis as their enlarged active surface and abundant adsorption sites facilitate metal-ion accumulation during the preconcentration step [290,291,292]. Such engineered architectures could also improve charge transfer and provide more electrochemically accessible sites, resulting in enhanced stripping signals and sensitive simultaneous determination of multiple metal ions [293,294].

Textured and nanostructured electrode interfaces are similarly attractive for food safety biosensing, particularly for rapid detection of pathogenic bacteria such as Salmonella and Staphylococcus aureus. Integration of high-surface-area carbon or gold nanostructures with aptamer-based recognition can improve receptor loading and target capture, enabling highly sensitive pathogen detection in complex food matrices [295,296,297,298]. Recent electrochemical platforms have additionally demonstrated simultaneous detection of multiple foodborne pathogens, highlighting the potential of structured electrode interfaces for portable and point-of-care food safety monitoring [299,300].

Overall, the increasing use of structured electrochemical interfaces demonstrates the important role of controlled surface architecture in improving the functionality of sensing platforms for biomedical, environmental, and food safety applications. The representative electrode structures, fabrication approaches, major application areas, and their potential relevance to VAM-based surface engineering are summarized in Table 6. Accordingly, Table 6 does not present all listed electrochemical interfaces as VAM-fabricated biosensors; rather, it distinguishes the level at which their demonstrated surface architectures can inform the future development of VAM-enabled sensing interfaces. The classification is based on whether the VAM connection is directly demonstrated, translational through a geometrically or functionally comparable micro-scale architecture, or prospective because substantial secondary processing and experimental validation remain necessary.

As evident from Table 6, the present connection between VAM and electrochemical biosensing is predominantly translational or prospective rather than directly demonstrated. The listed studies nevertheless provide useful evidence that controlled hierarchical, patterned, and porous interfaces can influence electrochemically active area, mass transport, charge transfer, and biomolecular interaction. Their significance to VAM therefore lies in identifying functional surface design principles that may be translated into deterministic mechanically generated textures, rather than demonstrating an already mature VAM-based biosensor manufacturing route. Direct validation will require comparative studies in which conventional and VAM-textured electrodes of identical material and surface chemistry are evaluated under the same electrochemical and biosensing conditions.

## 7. Challenges and Future Research Directions

Despite the demonstrated advantages of engineered micro and hierarchical textured electrodes, translating these concepts into VAM-enabled biosensors requires further advances in manufacturing control, functional integration, and biological validation. In particular, future research should establish direct relationships between VAM process parameters, resulting surface texture, tribological durability, and electrochemical response rather than extrapolating performance from conventionally textured or nanostructured electrodes. Recent developments in vibration texturing and electrochemical biosensing provide a foundation for addressing these challenges [246].

### 7.1. Process Scalability and Reproducibility

A major challenge in the practical implementation of VAM-based surface texturing is achieving uniform and reproducible texture characteristics when the process is scaled from laboratory specimens to large batches of functional electrodes. During repeated machining, factors such as progressive tool wear, fluctuations in vibration amplitude, positioning errors, and variations in machining conditions can alter the dimensions and distribution of the generated textures, potentially affecting their functional performance. To address these issues, future VAM systems should incorporate in-process monitoring and adaptive control strategies capable of detecting changes in texture formation and adjusting machining parameters accordingly. More importantly, acceptable manufacturing tolerances for texture depth, spacing, and density should be established and correlated with electrochemical performance. Such process–surface–function relationships will be essential for ensuring reproducibility and supporting the transition of VAM-generated functional surfaces toward scalable biosensor manufacturing [144,246].

These manufacturing advantages, however, remain dependent on maintaining a stable VAM process window, as during prolonged machining, progressive tool wear, variations in cutting forces and vibration amplitude, local heating, burr formation, residual stresses, surface contamination, and substrate deformation may introduce dimensional variation or gradually reduce texture fidelity. Their functional consequences may also differ. Changes in texture depth, edge geometry, subsurface integrity, or burr formation can alter contact pressure, lubricant retention, debris entrapment, friction, and wear, whereas surface contamination, uncontrolled roughness, coating damage, altered wettability, or variation in electrochemically active area may affect background current, interfacial impedance, and sensing reproducibility. These effects become particularly important for thin, coated, or mechanically compliant electrodes, where excessive mechanical loading may also compromise the coating–substrate interface. Therefore, the advantages of deterministic VAM texturing should be evaluated within a controlled processing window in which tool condition, vibration stability, texture fidelity, and surface integrity are adequately maintained.

### 7.2. Multi-Scale Hierarchical Texturing

A particularly promising direction in case of textured electrode surfaces is the development of hierarchical texture electrodes in which VAM-generated micro-textures serve as a controlled scaffold for secondary nano-scale structures. Subsequent anodization, electrodeposition, hydrothermal growth, or conformal thin-film deposition could introduce nano-scale functionality without sacrificing the deterministic geometry of the underlying micro-texture. Such micro–nano integration may provide independent control over lubricant/fluid retention, electrochemically active surface area, mass transport, and bioreceptor immobilization. However, systematic studies are required to determine whether these benefits originate from the micro-scale geometry, nano-scale functional layer, or their synergistic interaction.

In addition, the technological relevance of incorporating VAM into a hybrid fabrication route should be assessed by the specific contribution of the VAM-generated micro-scale architecture. VAM becomes particularly useful when precise control of texture depth, spacing, orientation, and repeatability is required to govern fluid retention, directional transport, contact behavior, or mechanical stability, while the subsequent nano-scale treatment provides the required electrochemical or biochemical functionality. Under such conditions, the two processing stages perform complementary rather than overlapping functions. Nevertheless, these functional advantages must be weighed against the additional equipment, tool wear, alignment requirements, processing time, and process complexity associated with VAM. Moreover, the VAM tool-path-based processing may limit throughput and large-area scalability compared with rapid laser scanning or batch-based fabrication techniques. Hence, future studies should compare hybrid VAM surfaces with equivalent single-scale textured surfaces in terms of functional performance, reproducibility, tool life, material compatibility, and scalability. These comparisons will justify whether the inclusion of VAM provides a meaningful technological advantage or not.

### 7.3. Artificial Intelligence in Process–Property Optimization

The interconnected relationship between VAM parameters, texture geometry, tribological behavior, and electrochemical response creates a multidimensional optimization problem which is difficult to resolve using conventional one-factor experimental approaches. Machine learning models could integrate machining parameters, surface metrology, friction/wear measurements, and electrochemical data to identify texture configurations which simultaneously satisfy mechanical and sensing requirements. A particularly valuable future direction would be physics-informed modeling, where established VAM kinematics and contact mechanics constrain data-driven predictions and reduce dependence on large experimental datasets. Ultimately, such frameworks could enable inverse design, in which the required biosensor functionality is specified first and suitable VAM conditions are predicted automatically.

However, the immediate barrier to AI-assisted inverse design is not model complexity, but the availability of reliable process–structure–property datasets. Such datasets should consistently link VAM parameters and material properties with the actual manufactured texture geometry, surface integrity, tribological behavior, and electrochemical response. At present, differences in materials, vibration conditions, texture characterization, and testing protocols can limit direct comparison between individual studies. Predictive models developed from small or heterogeneous datasets may therefore show limited transferability beyond the conditions used for training. Establishing standardized experimental datasets and reporting protocols should consequently precede the development of more complex inverse-design frameworks.

### 7.4. In Vivo Stability and Biocompatibility

For implantable and long-term biosensors, maintaining electrode functionality within the biological environment remains far more challenging as compared to achieving high sensitivity under controlled laboratory conditions. Biofouling, foreign-body responses, mechanical deformation, and progressive changes at the tissue–electrode interface can alter mass transport and electrochemical characteristics during prolonged operation. Future studies on VAM-textured electrodes should therefore combine electrochemical measurements with tribological durability, cytocompatibility, protein adsorption, and physiologically representative mechanical testing before proceeding to in vivo validation. Such an approach would clarify whether engineered textures retain their intended functionality throughout realistic service conditions rather than only during short-term electrochemical testing.

A further challenge is that biological validation cannot be separated from manufacturing and device preparation. Texture geometry and functional coatings must remain stable after sterilization, while repeated fabrication should produce electrodes with comparable surface characteristics and electrochemical responses. Sterilization-induced changes in surface chemistry, wettability, coating integrity, or immobilized biological components could alter the intended function even when the underlying texture remains geometrically unchanged. Therefore, long-term mechanical stability, resistance to biofouling, biocompatibility, sterilization compatibility, and manufacturing reproducibility should be established together before meaningful in vivo validation of VAM-textured biosensor electrodes.

### 7.5. Sustainable and Green Manufacturing

Sustainability should also be considered when comparing the VAM process against laser, lithographic, and chemically assisted surface texturing techniques. As a mechanically driven process, VAM offers opportunities to generate functional textures while reducing dependence on masks, chemical etchants, and complex multi-stage patterning routes; however, its environmental advantage should be demonstrated quantitatively rather than assumed. Future investigations should therefore evaluate energy consumption, tool life, material waste, and the feasibility of dry or minimum-quantity-lubrication conditions alongside texture quality and sensing performance. A life-cycle-based comparison of VAM with competing texturing technologies would provide a stronger basis for identifying genuinely sustainable biosensor manufacturing routes.

## 8. Conclusions

The present manuscript examined vibration-assisted machining as a manufacturing route for translating selected biological surface design principles into deterministic functional textures. The analysis indicates that the value of VAM extends beyond the generation of surface roughness or periodic machining marks. Through controlled vibration kinematics, the process can create directional, overlapping, multi-scale, and hierarchical architectures that reproduce functional principles observed in natural surfaces, including fluid guidance, contact-area regulation, lubricant retention, debris accommodation, wettability control, and interfacial transport. These characteristics provide a useful bridge between biomimetic surface engineering, tribology, and electrochemical biosensing. The principal conclusions are summarized as follows:

- Biological surfaces provide transferable structure–function principles for engineering textures, particularly through directional organization, hierarchical architecture, controlled wettability, adhesion, and fluid management.
- VAM offers a deterministic route for translating several of these principles into manufacturable dimples, grooves, crosshatch patterns, fish-scale structures, and hierarchical textures while preserving surface integrity.
- Texture function depends on the combined effects of geometry, hierarchy, orientation, spacing, and density, which jointly regulate contact mechanics, lubrication, fluid transport, wettability, biofouling, and electrochemical accessibility.
- Hierarchical micro–nano-architectures provide the strongest connection between biomimetic design and biosensing because micro-scale structures can provide mechanical and fluid management functions while nano-scale features support electrochemical activity and biomolecular interactions.
- Future work should move from qualitative biological inspiration toward quantitative bio-inspired design rules linking natural structure, engineered geometry, VAM parameters, tribological durability, and sensing performance, supported by AI-assisted inverse design, physiological testing, and scalable manufacturing.

The principal opportunity was therefore not simply to reproduce the appearance of biological surfaces, but to identify the underlying structure–function relationships and translate them into manufacturable engineering designs. Combining biomimetic design principles with the geometric control of VAM and subsequent nano-scale functionalization could provide a practical route toward multifunctional interfaces that integrate mechanical durability, controlled biological interactions, and electrochemical sensing. Establishing this biology–design–manufacturing–function pathway should be a central objective of future research on VAM-enabled biomimetic surfaces.

## Figures and Tables

**Table 1:** Influence of VAM processing parameters on texture characteristics and their functional implications.

VAM Processing Parameter	Main Texture Characteristics Affected	Tribological Relevance	Potential Electrochemical Relevance	Reference
Vibration amplitude (A)	Texture depth, feature height, aspect ratio	Lubricant storage and hydrodynamic pressure generation	Electrolyte accessibility and available surface area	[25,44]
Vibration frequency (f)	Texture periodicity, pitch, feature density	Distribution of lubricant and repeated hydrodynamic effects	Spatial distribution of the textured active interface	[45,46]
Feed rate/feed per tooth (fz)	Feature spacing, overlap, texture density	Lubricant distribution and debris accommodation	Fluid and analyte transport between textured regions	[45,46]
Spindle/cutting speed	Texture periodicity, continuity, and spatial distribution	Uniformity of texture–contact interaction	Uniformity of the textured electrode interface	[45,46]
Depth of cut (ap)	Feature depth and overall dimensions	Lubricant storage capacity and load-bearing characteristics	Geometrically accessible surface area	[25,44]
Tool geometry/cutting edge radius	Feature width, edge profile, local curvature	Contact stress and texture integrity	Accessibility of the textured interface	[47,48,49]
Vibration direction/phase relationship	Texture shape, orientation, anisotropy	Directional or multidirectional lubricant transport	Directional electrolyte/analyte transport	[55,56,57]
Multi-pass processing	Crosshatched and hierarchical texture organization	Combined lubricant retention, debris trapping, and contact control	Potential increase in accessible area and transport pathways	—

**Note:** The electrochemical relevance presented in Table 1 represents the potential functional implications of VAM-controlled texture geometry rather than direct evidence of electrochemical performance following VAM, unless specifically demonstrated in the cited study.

**Table 2:** Surface texture parameters and their role.

Parameter	Tribological Role	Electrochemical Role	Typical Optimum Value
Aspect ratio	Lubricant retention, hydrodynamic pressure	Electrolyte retention, diffusion	0.1–0.3
Texture density	Load support, debris trapping	Active surface area	10–40%
Edge sharpness	Stress concentration	Current distribution	Rounded edges
Spatial arrangement	Lubricant flow	Mass transport	Periodic/stochastic
Orientation	Friction anisotropy	Electrolyte flow direction	Application-dependent

**Table 3:** Comparison of different surface texturing techniques mainly used for functional tribological surfaces and electrochemical biosensor electrodes.

Surface Texturing Method	Achievable Texture Geometry	Surface Integrity	Suitable Materials	Major Advantages	Major Limitations
Vibration-assisted machining (VAM)	Dimples, grooves, crosshatch, waffle, hierarchical textures	Excellent; negligible HAZ, smooth edges, low residual stress	Metals, polymers, composites	High geometric accuracy, excellent surface finish, suitable for functional textures	Limited commercial availability and relatively low productivity
Laser surface texturing (LST)	Dimples, grooves, grids, biomimetic textures	Moderate; possible heat-affected zone (HAZ), recast layer, burr formation	Metals, ceramics, polymers, composites	High flexibility, non-contact process, complex texture generation	Thermal damage and high equipment cost
Electrochemical micromachining (ECM)	Micro-dimples, grooves, cavities	Excellent; stress-free and burr-free	Electrically conductive materials	No thermal damage, excellent surface integrity	Restricted to conductive materials and electrolyte management
Electrical discharge machining (EDM)	Dimples, grooves, cavities	Moderate; recast layer and microcracks may form	Electrically conductive materials	Capable of machining hard materials and complex geometries	Thermal damage, recast layer, relatively low surface quality
Focused ion beam (FIB)	Arbitrary nano/micro-textures	Excellent	Nearly all materials	Ultra-high precision and nano-scale fabrication	Extremely low material removal rate and very high cost
Photolithography + etching	Regular microarrays, pillars, channels	Excellent on planar substrates	Silicon, glass, polymers	High repeatability and batch fabrication	Limited to planar substrates and cleanroom processing

**Table 4:** Biological inspiration and translation into VAM-compatible functional surface textures.

Biological Model	Natural Surface Feature	Biological/Functional Principle	Engineering Analog	VAM-Compatible Texture	Relevance to Tribology and Biosensing
Fish scales	Overlapping curved scales	Protection, directional fluid guidance, reduced drag	Overlapping scallops	Fish-scale/overlapping texture	Lubricant or electrolyte guidance; debris accommodation; controlled mass transport [4,79]
Shark skin	Anisotropic placoid/riblet organization	Near-wall flow control and drag reduction	Directional micro-grooves/riblets	Linear, oblique, or staggered grooves	Directional lubrication and analyte transport; shear reduction [80]
Lotus leaf	Hierarchical micro-/nano-roughness	Water repellency and self-cleaning	Dual-scale hierarchical texture	VAM micro-texture + nano-scale functionalization	Wettability control, antifouling, controlled biomolecular interaction [77]
Gecko foot	Hierarchical setae/fibrillar structures	High and controllable adhesion	Micro/nanopillar or fibrillar arrays	VAM-defined microarrays + secondary nanostructures	Contact-area and adhesion control; potential tuning of immobilization interfaces [78]
Morpho butterfly wing	Multi-scale anisotropic ridges/roughness	Direction-dependent wettability	Periodic anisotropic ridges	Wave, groove, or crosshatch textures	Directional wetting and passive fluid/analyte guidance [5]
Hierarchical biological interfaces	Features organized across multiple length scales	Multifunctionality through scale-dependent mechanisms	Micro-texture carrying nano-scale functional layer	VAM + anodization/electrodeposition/ALD/nanomaterials	Mechanical durability plus high EASA, charge transfer, and bioreceptor loading

**Table 5:** Tribological behavior of various VAM-generated surface textures with dominant lubrication regimes.

Texture Type	Depth (µm)	Texture Density (%)	Dominant Lubrication Regime	Reference
Micro-dimples	5–20	10–25	Hydrodynamic	[102,128]
Linear grooves	3–15	15–30	Mixed	[43,129]
Crosshatch pattern	5–25	20–35	Boundary	[130,131]
Hierarchical (VAM)	2–30	10–40	All regimes	[20,132,133]
Fish-scale	3–18	15–28	Mixed/hydrodynamic	[4]
Pillar array	8–30	15–35	Boundary	[102]

**Table 6:** Representative structured electrochemical sensing interfaces, fabrication strategies, and their relevance to VAM-enabled surface engineering.

Electrode/Sensing Material	Surface Architecture	Fabrication/Modification Route	Application Category	Year	Evidence of VAM Relevance	Reference
Au/MXene/GCE	Hierarchical Au/MXene	Electrodeposition	Metabolic sensing	2024	Translational—hierarchical architecture	[301]
Au/Ni-MOF/GCE	Hierarchical porous structure	Electrochemical assembly	Metabolic/oxidative sensing	2024	Translational—hierarchical/porous architecture	[302]
BN nanosheet electrode	2D nanostructured interface	Nanosheet modification	Cancer biomarkers	2024	Prospective—nano-scale functionalization required	[303]
Au nanostructured array	Nanostructured Au	Electroplating	Genetic biomarkers	2025	Translational—patterned surface architecture	[264]
MgCo_2_O_4_/CNT/GCE	Porous CNT/oxide hybrid	Hydrothermal modification	Neurochemical sensing	2025	Prospective—secondary nanostructuring required	[304]
Bi/porous graphene	Hierarchical porous interface	Electrodeposition	Environmental monitoring	2024	Prospective—porous functional layer requires secondary processing	[291]
Nanostructured aptasensor	Nanostructured interface	Aptamer functionalization	Food safety	2025	Prospective—biochemical functionalization required	[305]
Laser-engraved graphene/AuNP	Porous hierarchical structure	Laser engraving	Wearable biosensing	2026	Translational—hierarchical textured architecture	[306]

Note: “Direct VAM evidence” refers to a functional surface experimentally produced by VAM or a directly related vibration-assisted texturing process and subsequently evaluated for the relevant function. “Translational” indicates that the reported electrochemical function was demonstrated using a non-VAM fabrication route, but the principal micro-scale geometry or surface design principle has potential correspondence with VAM-generated textures. “Prospective” indicates that substantial secondary nanostructuring, chemical/biochemical functionalization, or direct experimental validation would be required before the architecture could be considered VAM-enabled. These classifications indicate the nature of the evidence and do not imply that the corresponding biosensor was experimentally fabricated by VAM.

## Data Availability

This publication is supported by multiple datasets which are available at locations cited in the ‘References’ section of this paper.
